# Sustained Effectiveness of Lanadelumab in Preventing Hereditary Angioedema Attacks: The ENABLE Study

**DOI:** 10.1002/clt2.70190

**Published:** 2026-08-06

**Authors:** Andrea Zanichelli, Walter A. Wuillemin, Markus Magerl, Andreas Recke, Mauro Cancian, Emel Aygören‐Pürsün, Ramón Lleonart Bellfill, Aharon Kessel, Tamar Kinaciyan, Robin Lochbaum, Mona Al‐Ahmad, Irmgard Andresen, Maureen Watt, Natalie Khutoryansky, Daniel N. Castaner, Inmaculada Martinez‐Saguer

**Affiliations:** ^1^ Operative Unit of Medicine Angioedema Center IRCCS Policlinico San Donato, San Donato Milanese Milan Italy; ^2^ Dipartimento di Scienze Biomediche per la Salute University of Milan Milan Italy; ^3^ Luzerner Kantonsspital Lucerne and University of Berne Berne Switzerland; ^4^ Charité‐Universitätsmedizin Berlin (corporate Member of Freie Universität Berlin and Humboldt‐Universität zu Berlin) Berlin Germany; ^5^ Fraunhofer Institute for Translational Medicine and Pharmacology ITMP IA Berlin Germany; ^6^ University of Lübeck Lübeck Germany; ^7^ University Hospital of Padua Padua Italy; ^8^ University Hospital Frankfurt Goethe University Frankfurt Frankfurt Germany; ^9^ Allergology Department Hospital Universitari de Bellvitge Institut de Recerca IDIBELL L'Hospitalet de Llobregat Barcelona Spain; ^10^ Bnai Zion Medical Centre Technion Faculty of Medicine Haifa Israel; ^11^ Department of Dermatology Medical University of Vienna Vienna Austria; ^12^ Ulm University Medical Center Ulm Germany; ^13^ Microbiology Department College of Medicine Kuwait University Kuwait City Kuwait; ^14^ Takeda Pharmaceuticals International AG Zurich Switzerland; ^15^ Takeda Development Center Americas Inc. Lexington Massachusetts USA; ^16^ HZRM Hemophilia Center Rhine Main Frankfurt/Main Germany

**Keywords:** effectiveness, hereditary angioedema, lanadelumab, long‐term prophylaxis, safety

## Abstract

**Background:**

Lanadelumab has been approved for hereditary angioedema (HAE) long‐term prophylaxis since 2018. The Phase 4, prospective ENABLE Study (NCT04130191) evaluated the long‐term effectiveness and safety of lanadelumab in clinical practice across Europe and the Middle East.

**Methods:**

Patients with HAE aged ≥ 12 years initiating lanadelumab treatment (300 mg every 2 weeks) per approved product labeling were recruited from Austria, Germany, Israel, Italy, Kuwait, Spain, and Switzerland and followed for up to 36 months (± 30 days). The primary objective was to evaluate lanadelumab effectiveness for HAE attack prevention. Safety and patient‐reported health‐related quality of life (HRQoL) were also evaluated.

**Results:**

Outcomes were analyzed in 138 patients (mean [range] age: 41.0 [14–79] years; 62.3% female; 92.0% HAE‐C1INH‐Type1), of whom > 60% extended dosing intervals by Month 12. Over a mean ± SD treatment duration of 28.6 ± 9.8 months, mean ± SD patient‐reported HAE attack rate decreased from 3.88 ± 3.43 attacks/month pre‐lanadelumab to 0.30 ± 0.53 attacks/month (mean decrease, 84% [median 96%]). The incidence‐rate ratio was 0.07 (95% CI: 0.06–0.10), reflecting a 93% reduction from modeled pre‐lanadelumab rates. Overall, 95/138 patients reported treatment‐emergent adverse events (TEAEs); most were unrelated to lanadelumab (82.3%). Of the 17.7% of TEAEs considered treatment‐related, injection‐site reactions, headache, fatigue, and asthenia occurred in > 1 patient. Before lanadelumab initiation, most patients reported moderate‐to‐large HRQoL impairments; clinically meaningful improvements were observed 1 month after lanadelumab initiation and sustained throughout the study.

**Conclusions:**

Real‐world data from ENABLE demonstrated long‐term effectiveness of lanadelumab in patients with HAE aged ≥ 12 years and a safety profile consistent with previous clinical studies.

## Graphical abstract

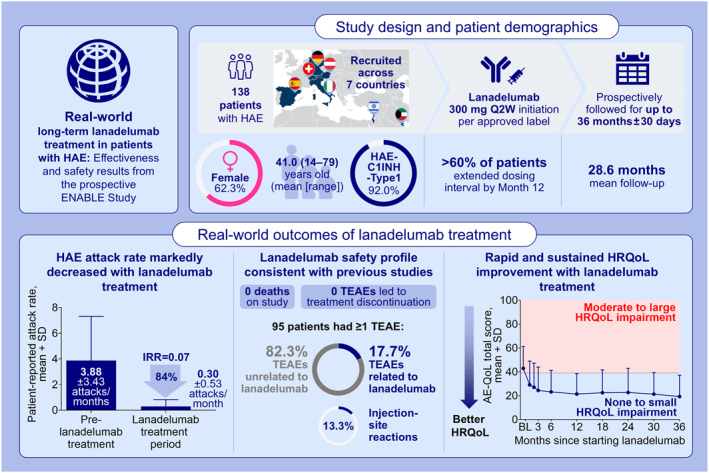

## 1 Introduction

Hereditary angioedema (HAE) is a rare genetic disorder (prevalence 1:50,000–1:100,000) that manifests as recurrent, unpredictable cutaneous and/or subcutaneous swelling attacks. These can occur anywhere in the body, including the skin, abdomen, and upper respiratory tract [1]. Laryngeal attacks can be fatal due to the risk of asphyxiation [2]. The frequency, severity, and anatomical location of HAE attacks varies significantly between patients [3]; this unpredictability often heightens anxiety and adds to impaired health‐related quality of life (HRQoL) [4, 5].

Most HAE cases are caused by C1 inhibitor (C1INH) deficiency, either due to diminished levels of circulating C1INH (HAE‐C1INH‐Type1) or dysfunctional C1INH (HAE‐C1INH‐Type2) [1]. Significant diagnostic delays are prevalent due to the rarity of HAE and inadequate awareness in the broader medical community [6]. In a recent systematic review that evaluated data from > 10,000 patients with HAE, mean and median diagnostic delay ranged between 3.9 and 26 years [7].

On‐demand therapies are recommended for treating HAE attacks [8, 9]. Current international guidelines recommend evaluating patients regularly for long‐term prophylaxis (LTP), with the goal to achieve complete disease control and normalization of their lives [8, 9]. During evaluations for LTP treatment, patients' preferences should be considered alongside disease activity and burden [9].

Lanadelumab is a fully human monoclonal antibody plasma kallikrein inhibitor approved for HAE LTP in multiple regions, including Europe, North and South America; in many countries, it is the only LTP therapy approved for patients as young as 2 years [10, 11, 12, 13, 14, 15, 16]. Current guidelines, including the 2025 World Allergy Organization HAE guidelines, the International/Canadian HAE guidelines, as well as the international pediatric HAE guidelines, all recommend lanadelumab as first‐line LTP [9, 17, 18]. The safety and efficacy of subcutaneous lanadelumab treatment has been extensively evaluated in clinical studies across patients in different countries with diverse clinical profiles [19, 20, 21, 22, 23]. The recommended starting dose of lanadelumab is 300 mg every 2 weeks (Q2W) in adults and adolescents and, per the EU label, the dosing interval may be extended to every 4 weeks (Q4W) in patients who are stably attack‐free on Q2W treatment [13, 14, 15], which may improve convenience for some.

Since receiving FDA and EMA approvals in 2018, the effectiveness and tolerability of lanadelumab have been described in multiple reports of data collected during routine clinical practice by investigators across different regions [24, 25, 26, 27, 28, 29, 30, 31, 32]. The Phase 4, noninterventional EMPOWER Study prospectively evaluated 109 patients in the United States and Canada for up to 36 months who were either newly treated or established on lanadelumab [33], and the retrospective INTEGRATED Study included 198 patients across Europe who received lanadelumab for a median duration of 29 months [34]. We report final outcomes of ENABLE, a prospective study conducted to further evaluate long‐term effectiveness and safety of lanadelumab in real‐world clinical practice, and to assess utilization and treatment patterns.

## 2 Methods

### 2.1 Study Design and Population

ENABLE (NCT04130191) was a Phase 4, non‐interventional, prospective, multicenter cohort study conducted in Austria, Germany, Israel, Italy, Kuwait, Spain, and Switzerland that evaluated the real‐world effectiveness, tolerability, and safety of lanadelumab in patients with HAE (Supporting Information S1: Figure S1). Patients aged ≥ 12 years were enrolled following a discussion between the physician and patient to initiate lanadelumab, and followed for up to either 24 months ± 30 days (if enrolled on or after March 1, 2021) or 36 months ± 30 days (if enrolled before March 1, 2021). Eligible patients were required to have information on HAE attack‐related variables for 3 calendar months before enrollment, to have initiated lanadelumab according to approved product labeling, and be able to collect data using a mobile device. Patients who discontinued lanadelumab before Month 24 could continue the study with scheduled assessments.

The study protocol was approved by an independent institutional review board and site‐specific independent ethics committees, as applicable by local regulations. Patients or their legally authorized representatives provided written informed consent. The study was conducted in accordance with the International Conference on Harmonisation and Good Clinical Practice guidelines and the Declaration of Helsinki.

### 2.2 Study Endpoints and Assessments

The primary endpoint was the incidence rate ratio of on‐treatment patient‐reported HAE attacks after lanadelumab initiation versus the 3‐month pre‐lanadelumab baseline period. HAE attacks were self‐reported by patients using a mobile application‐based attack diary, and recorded by physicians using electronic case report forms (eCRFs) during patient visits. Other endpoints included on‐treatment attack characteristics, lanadelumab treatment patterns, patient‐reported outcomes (PROs), and safety. A post hoc stratification of lanadelumab treatment pattern by enrollment date (before vs. after March 1, 2021) was performed, as follow‐up data beyond Month 24 were largely contributed by patients enrolled before this date. Safety assessments included treatment‐emergent adverse events (TEAEs) and serious TEAEs captured by eCRFs, and were summarized by severity and relationship to lanadelumab treatment. PROs were assessed using the Angioedema Control Test (AECT) [35, 36], Angioedema Quality of Life (AE‐QoL) [37, 38], Fatigue Severity Scale (FSS) [39], Hospital Anxiety and Depression Scale (HADS) [40, 41], Work Productivity and Activity Impairment: General Health (WPAI‐GH) [42, 43], and Treatment Satisfaction Questionnaire for Medication‐9 (TSQM‐9) [44] (Supporting Information S1: Supplementary Methods).

### 2.3 Statistical Analysis

Safety outcomes were analyzed in the safety set (all enrolled patients who received ≥ 1 lanadelumab dose). Effectiveness outcomes were analyzed in the full analysis set (FAS), which included all patients in the safety set who had ≥ 1 post‐enrollment effectiveness outcome assessment. Observed HAE attack rates were calculated as mean (SD), median, and first and third quartiles (Q1, Q3) of number of attacks per month in FAS. Mean and median HAE attack rate reductions post‐lanadelumab initiation versus composite pre‐lanadelumab period were also calculated. Model‐estimated means and 95% confidence intervals (CIs) for HAE attack rates were calculated using a generalized linear model with a negative binomial distribution and a log link using log‐transformed observation period durations as an offset parameter. All statistical analyses were conducted using SAS version 9.4 (SAS Institute, Cary, NC, USA).

## 3 Results

### 3.1 Patient Disposition and Baseline Characteristics

The study enrolled 139 patients at 18 sites across 7 countries, 138 were included in both safety set and FAS (Supporting Information S1: Figure S2; Table 1). Three sites recruited ∼56% of patients (39, 23, and 15 patients in single German, Italian, and Swiss sites, respectively). Two patients completed the study beyond the Month 36 visit window (by 3 and 8 days, respectively). Of the 31 patients who discontinued the study, the most common reason for discontinuation was withdrawal by the patient with no other reason recorded (*n* = 14; 10.1%), followed by physician's decision (*n* = 5; 3.6%), lost to follow‐up (*n* = 5; 3.6%), pregnancy (*n* = 2; 1.4%), and other (*n* = 5; 3.6%). No discontinuations due to TEAEs were recorded. The mean follow‐up in patients who discontinued was 16.0 months. In the 3 months preceding enrollment, 111 patients experienced ≥ 4 attacks; however, information on attack location and treatment were missing for most attacks. Prior LTP use was reported in 38 patients; 5 discontinued LTP due to lack of efficacy in the 3 months preceding lanadelumab initiation. In 9 patients who had received prior LTP with IV plasma‐derived C1INH, there was a short period (≤ 10 days in 7 patients, ∼8 weeks in 2 patients) when both LTPs were administered after lanadelumab initiation and before plasma‐derived C1INH was discontinued. Two patients tapered attenuated androgens (over ∼1 month and ∼5 months, respectively) after initiating lanadelumab.

**TABLE 1:** Patient demographics and baseline disease characteristics.

Characteristics	Total (*N* = 138)^a^
Age at enrollment, years	
Mean (SD); median (range)	41.0 (14.4); 42.0 (14–79)
Age at enrollment category, *n* (%)	
12 to < 18 years	8 (5.8)
18 to < 40 years	55 (39.9)
40 to < 65 years	68 (49.3)
≥ 65 years	7 (5.1)
Sex, *n* (%)	
Male/Female	52 (37.7)/86 (62.3)
Race, *n* (%)	
White	137 (99.3)
Black or African American	1 (0.7)
Ethnicity, *n* (%)	
Latino or Hispanic	3 (2.2)
Not Latino or Hispanic	135 (97.8)
BMI,^b^ kg/m^2^	
Mean (SD); median (range)	27.2 (6.6); 26.6 (17.3–50.5)
Age at onset of HAE symptoms, years	
Mean (SD); median (range)	10.1 (9.7); 7.0 (0–69)
Age at diagnosis of HAE, years	
Mean (SD); median (range)	18.9 (13.6); 17.0 (0–71)
Delay in HAE diagnosis,^c^ years	
Mean (SD); median (range)	8.8 (11.2); 4.2 (−16–53)
HAE type, *n* (%)	
HAE‐C1INH‐Type1	127 (92.0)
HAE‐C1INH‐Type2	10 (7.2)
HAE‐nC1INH	1 (0.7)
HAE characteristics in the last 3 months prior to screening	
Attack rate, attacks/4 weeks, *n* = 134	
Mean (SD); median (range)	4.1 (3.5); 3.0 (0.0–16.7)
Attack rate category, *n* (%)	
0 to < 1 attacks/4 weeks	18 (13.0)
1 to < 2 attack/4 weeks	26 (18.8)
2 to < 3 attacks/4 weeks	15 (10.9)
≥ 3 attacks/4 weeks	75 (54.3)
Missing	4 (2.9)
Severe attack rate, attacks/4 weeks, *n* = 131	
Mean (SD); median (range)	1.0 (2.0); 0.3 (0.0–13.3)
Severe attack rate category, *n* (%)	
0 to < 1 attacks/4 weeks	95 (68.8)
1 to < 2 attack/4 weeks	16 (11.6)
2 to < 3 attacks/4 weeks	7 (5.1)
≥ 3 attacks/4 weeks	13 (9.4)
Missing	7 (5.1)
Pre‐enrollment LTP therapy, *n* (%)^d^	38 (27.5)
Plasma‐derived C1INH replacement therapy, IV	22 (15.9)
Plasma‐derived C1INH replacement therapy, SC	7 (5.1)
Attenuated androgens (e.g., danazol, oxandrolone, stanozolol)	9 (6.5)

Abbreviations: BMI, body mass index; C1INH, C1 inhibitor; HAE, hereditary angioedema; IV, intravenous; SC, subcutaneous; SD, standard deviation.

^a^ Full analysis set.

^b^ BMI information missing for 15 patients.

^c^ Time from HAE symptom onset to HAE diagnosis (date of HAE diagnosis−date of HAE symptom onset + 1)/365.25. In some patients, diagnosis of HAE preceded manifestation/identification of first symptoms; in these cases, calculations for time from HAE symptom onset to diagnosis yielded negative values.

^d^ During the 3 months prior to initiation of lanadelumab.

### 3.2 Lanadelumab Exposure

**FIGURE 1:**
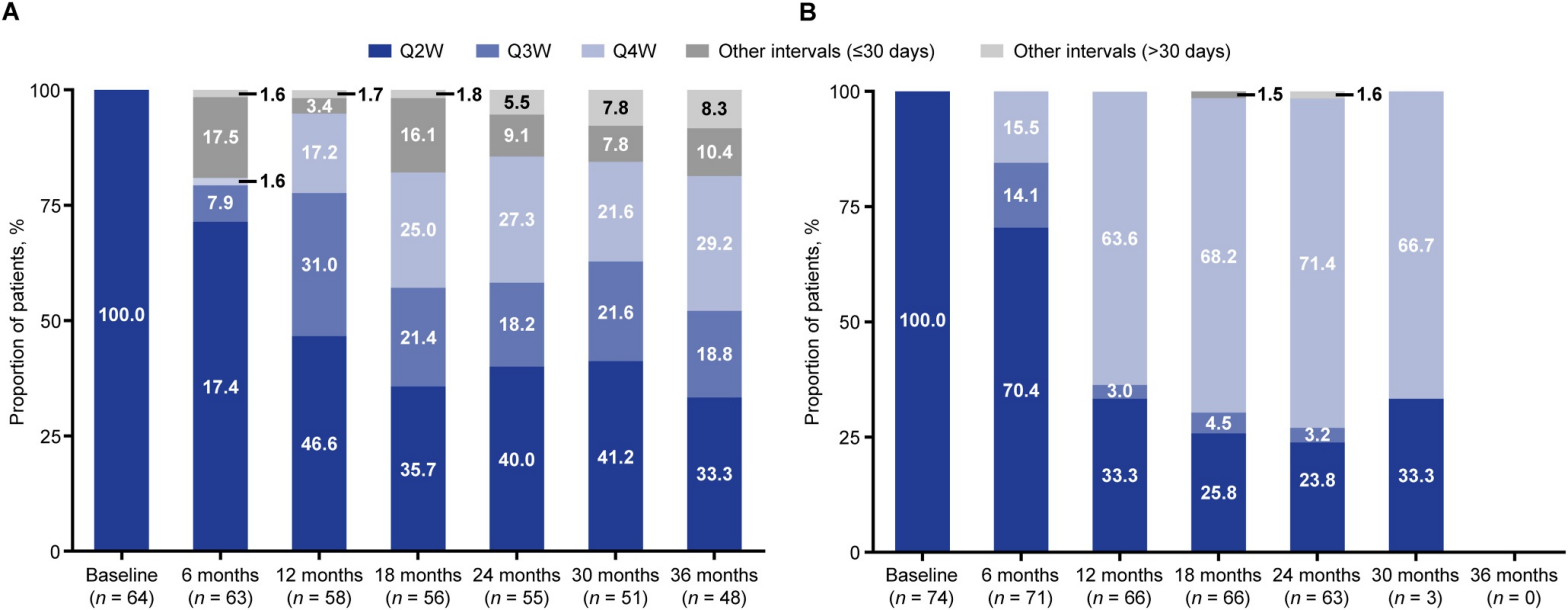
Lanadelumab treatment dosing during the study period in (A) patients who enrolled on or before March 1, 2021 and (B) patients who enrolled after March 1, 2021. Dosing intervals were between 12 and 16 days for Q2W, between 19 and 23 days for Q3W, and between 26 and 30 days for Q4W. All other dosing intervals were grouped as either ≤ 30 days or > 30 days. Q2W, every 2 weeks; Q3W, every 3 weeks; Q4W, every 4 weeks.

Lanadelumab treatment duration ranged between 57 and 1133 days (Q1–Q3: 714–1087), with a mean (SD) of 800.4 (273.8) days. All patients received a starting dose of 300 mg lanadelumab Q2W; by Months 6 and 12, 28.4% and 60.5% had extended dosing intervals, respectively. Post hoc analysis revealed distinct treatment patterns between patients who enrolled before and those who enrolled after March 1, 2021; most patients in the latter cohort extended dosing interval to Q4W by Month 12, whereas dosing intervals among the former were more varied (Figure 1). In total, 95 (68.8%) patients had ≥ 1 dosing interval change during follow‐up. The median time on Q2W dosing before interval extension was 191 days (95% CI, 183–300). The observed mean (range) number of lanadelumab doses before any treatment modification was 13.2 (1–68).

### 3.3 Effectiveness

**FIGURE 2:**
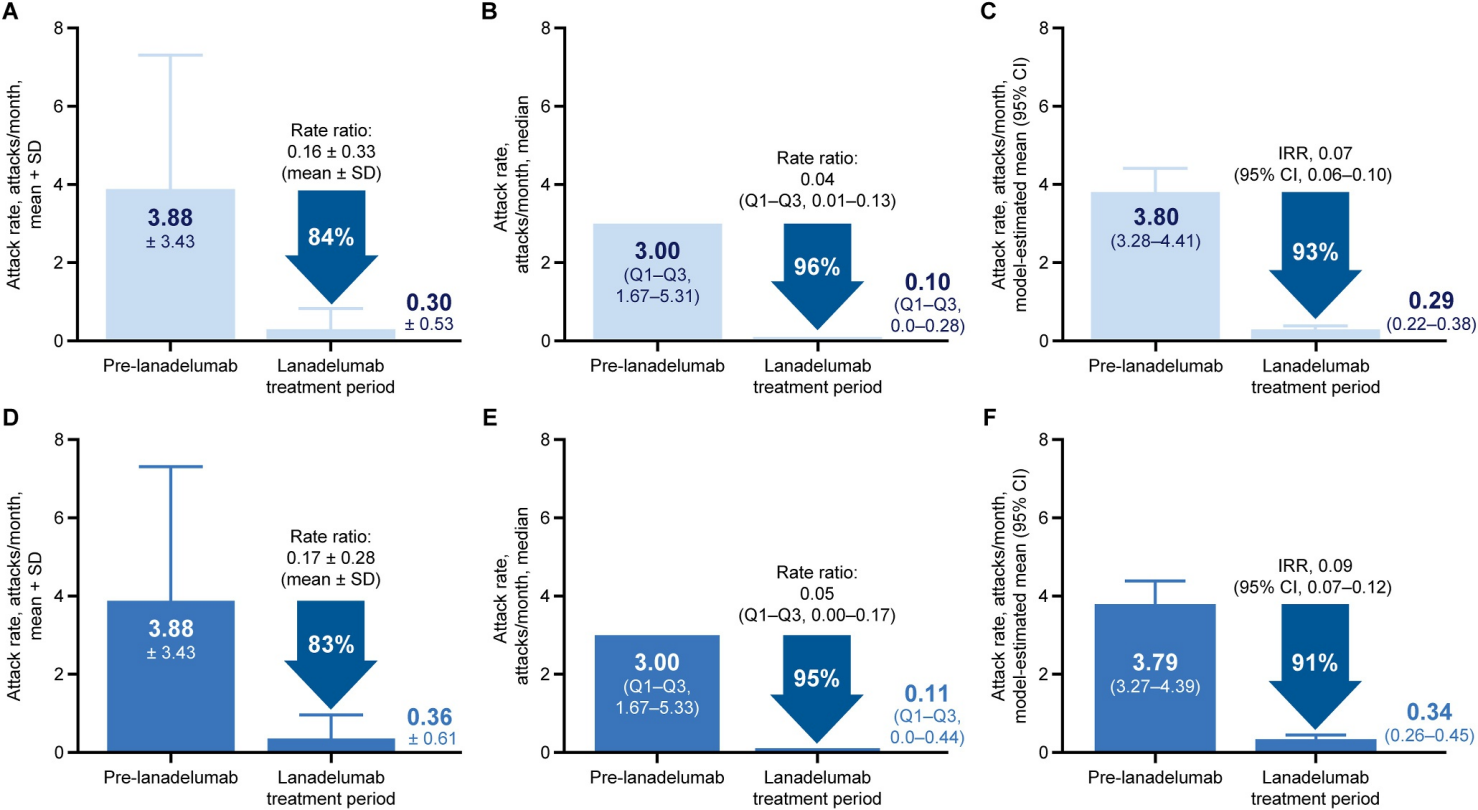
HAE attack rates before and after lanadelumab initiation. Patient‐reported (A) mean, (B) median, and (C) model‐estimated mean attack rate. Physician‐reported (D) mean, (E) median, and (F) model‐estimated mean attack rate. CI, confidence interval; HAE, hereditary angioedema; IRR, Incidence rate ratio; Q1, first quartile; Q3, third quartile; SD, standard deviation.

The mean (SD) patient‐reported observed HAE attack rate decreased by 84% from 3.88 (3.43) attacks/month pre‐lanadelumab to 0.30 (0.53) attacks/month on treatment (Figure 2A). The median attack rate decreased by 96%, from 3.0 attacks/month pre‐lanadelumab to 0.1 attacks/month on treatment (Figure 2B). Modeled attack rate based on patient‐reported data (Figure 2C) and attack rates based on physician‐reported information (Figure 2D–F) showed similar trends. Notably, 82 (59.4%) of the 138 patients were attack‐free for any 4‐week period following lanadelumab initiation, contrasting with the baseline period in which only 12 (8.7%) patients were attack‐free for a total of 1 month.

Data on attack characteristics before lanadelumab initiation were limited; however, information on attack severity and treatment was available for most patient‐reported and physician‐reported attacks during the treatment period (Table 2). Of the attacks with available information during lanadelumab treatment, ≥ 80% were mild or moderate and ∼70% were treated, mostly with C1INH or icatibant.

**TABLE 2:** HAE attack characteristics before and after lanadelumab initiation.

	Patient diary record		Physician‐reported	
	Pre‐lanadelumab (*n* = 132)^a^	Lanadelumab treatment period (*n* = 101)^a^	Pre‐lanadelumab (*n* = 132)^a^	Lanadelumab treatment period (*n* = 99)^a^
Duration of observation period, days (SD)	86.8 (7.4)	800.4 (273.8)	86.8 (7.4)	800.4 (273.8)
Number of attacks	1656	1011	1660	1205
Attacks affecting body parts, *n* (%)^b^				
Unknown/missing	1635	28	634	871
Attacks with information available	21	983	1026	334
Peripheral	8 (0.5)	421 (41.6)	565 (34.0)	167 (13.9)
Airways or upper airway	1 (0.1)	121 (12.0)	58 (3.5)	22 (1.8)
Abdomen	11 (0.7)	572 (56.6)	517 (31.1)	173 (14.4)
Other	4 (0.2)	119 (11.8)	14 (0.8)	12 (1.0)
Attack severity, *n* (%)^a^				
Unknown/missing	1635	28	689	13
Attacks with information available	21	983	971	1192
Grade 1 (mild)	8 (0.5)	335 (33.1)	265 (16.0)	407 (33.8)
Grade 2 (moderate)	10 (0.6)	509 (50.3)	522 (31.4)	656 (54.4)
Grade 3 (severe)	3 (0.2)	139 (13.7)	184 (11.1)	129 (10.7)
Grade 4 (life‐threatening)	0	0	0	0
Treatment received for the attack, *n* (%)^a^				
Unknown/missing	1635	28	600	1
Attacks with information available	21	983	1060	1204
Any treatment	16 (1.0)	680 (67.3)	984 (59.3)	879 (72.9)
Plasma‐derived C1INH replacement therapy	14 (0.8)	451 (45.9)	725 (43.7)	580 (48.1)
Recombinant human C1INH replacement therapy	0	6 (0.6)	1 (0.1)	4 (0.3)
Icatibant	2 (0.1)	250 (44.6)	139 (8.4)	295 (24.5)
Other	0	19 (1.9)	2 (0.1)	8 (0.7)
Unknown treatment	0	0	135 (8.1)	30 (2.5)
No treatment	5 (0.3)	303 (30.0)	76 (4.6)	325 (27.0)

Abbreviations: C1INH, C1 inhibitor; SD, standard deviation.

^a^ Number of patients with attack information available.

^b^ Denominator is the total number of attacks.

### 3.4 Patient‐Reported Outcomes

**FIGURE 3:**
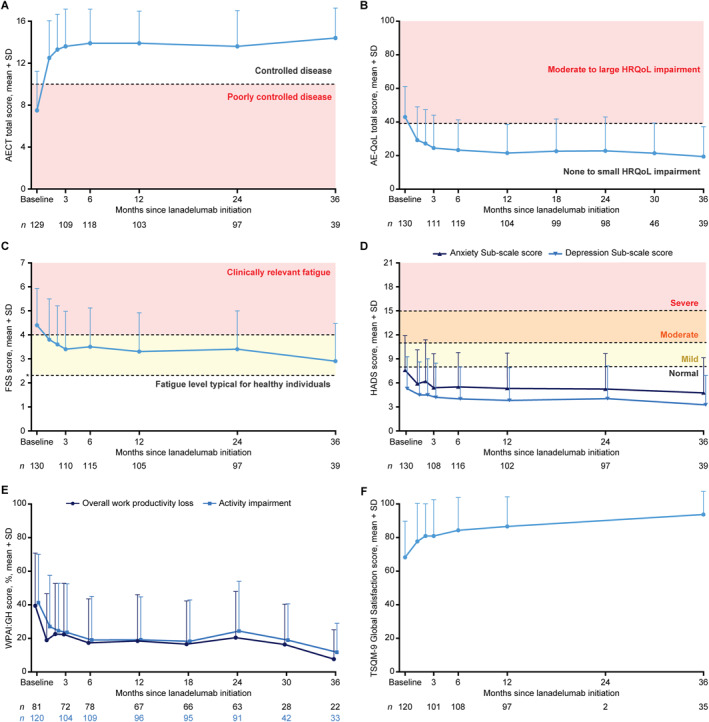
Patient‐reported outcomes over time for (A) AECT total score (score ranges from 0 to 16 with higher score indicating perception of better disease control; AECT score < 10, perception of poorly controlled disease, AECT ≥ 10, perception of well‐controlled disease [36]; a change of 3 points is considered the minimal clinically important difference) [45], (B) AE‐QoL total score (score is transformed to a scale of 0–100 with higher score indicating greater impairment: score of 0–23 indicates no effect, 24–38 a small effect, and ≥ 39 a moderate‐to‐large effect of recurrent angioedema on patients' HRQoL; a change of 6 points is considered the minimal clinically important difference [37, 38]), (C) FSS score (score ranges from 1 to 7 with higher score indicating worse fatigue; a FSS score of 2.3 [indicated with “*”] is typical for healthy individuals and a score of ≥ 4 indicates clinically relevant fatigue [39, 46]), (D) HADS subscale scores (scores range from 0 to 21 with higher scores indicating greater impairment: scores of 0–7 indicate normal levels of anxiety or depression, 8–10 mild, 11–14 moderate, and 15–21 severe anxiety or depression) [40, 41, 47], (E) WPAI:GH work productivity loss and activity impairment (scores are expressed as impairment percentages with higher scores indicating greater impairment and reduced productivity) [42, 43], and (F) TSQM‐9 global satisfaction score (score ranges from 0 to 100 with higher score indicating greater satisfaction) [44]; data for Month 24 not shown as most data were not captured due to deployment issue. All outcomes are reported as mean + SD. Baseline defined as the last observed value taken at enrollment assessment before lanadelumab initiation. Values for each patient‐reported outcome measure are reported in Supporting Information S1: Tables S1–S6. AECT, Angioedema Control Test; AE‐QoL, Angioedema Quality of Life; FSS, Fatigue Severity Scale; HADS, Hospital Anxiety and Depression Scale; TSQM‐9, Treatment Satisfaction Questionnaire for Medication‐9; WPAI: GH, Work Productivity and Activity Impairment: General Health.

The mean (SD) AECT total score at baseline was 7.5 (3.7), indicating patient perception of poorly controlled disease [35, 36]. By the first assessment at Month 1, ∼80% (81/103) of patients had AECT total scores ≥ 10 (indicating perception of controlled disease); the mean (SD) AECT total score increased to 12.5 (3.6) (Supporting Information S1: Figure S3A), continuing to increase over time on treatment (Figure 3A; Supporting Information S1: Table S1). After Month 6, the median AECT total score was 16 (maximum score) at most time points (Supporting Information S1: Figure S3B).

At baseline, the mean and median AE‐QoL total scores were 42.9 and 41.2, respectively, indicating moderate‐to‐large HRQoL impairments (represented by scores of ≥ 39 [37, 38]). Clinically meaningful HRQoL improvement (≥ 6‐point decrease [37, 38]) was observed at the first timepoint after lanadelumab initiation, with mean and median AE‐QoL total score decreasing to 29.1 and 22.1 at Month 1, respectively (Supporting Information S1: Figure S4A–B). HRQoL improvement continued over time, as indicated by further decreases in AE‐QoL total score (Figure 3B; Supporting Information S1: Table S2) and domain scores (Supporting Information S1: Figure S5A–D).

Following lanadelumab initiation, mean and median FSS total scores decreased from 4.4 and 4.5 at baseline (indicative of clinically relevant fatigue [39, 46]) to 3.8 and 3.8 at Month 1, respectively (Supporting Information S1: Figure S6), and continued to decline further during the study (Figure 3C; Supporting Information S1: Table S3). Similarly, 1 month after initiating lanadelumab, the mean and median HADS Anxiety Subscale Score decreased from 7.6 and 8.0 at baseline (indicative of normal to mild anxiety [41]) to 5.9 and 5.0, respectively (Supporting Information S1: Figure S7), and continued to decrease throughout the study (Figure 3D; Supporting Information S1: Table S4). Among adult patients who responded to the questionnaire, the mean scores for overall work productivity loss, absenteeism, presenteeism, and activity impairment at baseline were 39.5%, 10.6%, 35.7%, and 41.3%, respectively. One month after initiating lanadelumab, the corresponding mean scores decreased to 19.0%, 4.8%, 17.8%, and 27.1% and remained low until the end of the study (Figure 3E; Supporting Information S1: Table S5; Figures S8–S9).

In line with other PROs, treatment satisfaction improved substantially following lanadelumab initiation, the mean (SD) TSQM‐9 global satisfaction score increased from 68.2 (21.5) at baseline to 77.7 (22.6) at Month 1 (Supporting Information S1: Figure S10), reaching 86.6 (17.7) by Month 12 (Figure 3F; Supporting Information S1: Table S6). Similar trends were seen for both effectiveness and convenience scores (Supporting Information S1: Figure S11).

### 3.5 Safety and Tolerability

In total, 474 non‐HAE attack‐related TEAE events were experienced by 95 (68.8%) patients (Supporting Information S1: Table S7). Most commonly reported TEAEs were infection/infestation‐type events (108 events in 59/138 [42.8%] patients). No TEAEs were fatal or led to discontinuation of lanadelumab treatment or the study. Of the 84 (17.7%) events in 25 (18.1%) patients considered lanadelumab treatment‐related, all but 1 were mild (71/84 events in 18 patients) or moderate (12 events in 6 patients) and non‐serious. One serious, severe treatment‐related TEAE of laryngeal edema was reported in 1 patient; the investigator confirmed the patient was not taking lanadelumab at the time of the event and attributed relatedness to temporary discontinuation of lanadelumab by the patient for a period of ∼3.5 months. Injection‐site reactions (ISRs) were the most frequently reported treatment‐related TEAEs (63 events, 13.3% of all TEAEs), but were reported in the minority of patients (16/138 [11.6%]; Supporting Information S1: Figure S12); patients most commonly reported erythema (5.1%) or pain (4.3%). Other reported TEAEs considered treatment‐related by the investigators were mild: asthenia, fatigue, and headache (2 events each), allergic dermatitis, back pain, chapped lips, discomfort, dysgeusia, dizziness, increased alanine aminotransferase, increased aspartate aminotransferase, intra‐abdominal hematoma, irregular menstruation, nausea, paresthesia, and tongue discomfort (all 1 event each), except for 1 TEAE of moderate migraine.

## 4 Discussion and Conclusion

ENABLE prospectively evaluated lanadelumab effectiveness and safety in 138 patients from 7 countries for up to 3 years. Most patients were highly symptomatic before lanadelumab initiation, and over a mean treatment duration of 2.2 years, model‐estimated patient‐reported attack rate decreased by 93% compared with the pre‐lanadelumab period. Clinically significant improvements in patient‐reported disease control and HRQoL were observed shortly after lanadelumab initiation and were maintained throughout the study period. No new safety signals were identified.

The mean attack‐rate reduction of 84% with lanadelumab observed in ENABLE (mean monthly attack rate: 3.88 [baseline] vs. 0.30 [on‐treatment]) was broadly similar to the mean attack‐rate reduction of 87.4% over a mean treatment duration of 2.5 years reported in the Phase 3 HELP OLE Study (mean monthly attack rate: 3.05 [baseline] vs. 0.25 [on‐treatment]) [22]. It is also consistent with 2 recently published noninterventional studies, EMPOWER [33] (85% decrease over mean 2.5‐year follow‐up; mean monthly attack rate in newly treated patients: 1.42 [baseline] vs. 0.02 [on‐treatment]) and INTEGRATED [34] (91.6% decrease over median 3.6‐year treatment duration; mean monthly attack rate: 3.0 [12 months pre‐lanadelumab] vs. 0.1 [on‐treatment]), despite different geographical coverage.

The relatively high pre‐enrollment attack rate observed in ENABLE may reflect infrequent LTP use, with ∼70% of patients reporting no LTP use before enrollment. Although guidelines recommend evaluating LTP need at every clinic visit [9], initiation thresholds vary with national regulations, patient preference, and clinician perspectives. In the Global Registry for HAE‐C1INH (follow‐up period: January 2018–August 2020), 30% of 1297 patients received LTP, most using attenuated androgens [48]. In the Phase 3 VANGUARD study (screening period: January 2021–June 2022), 33% of 64 patients with HAE‐C1INH used LTP in the 3 months before screening, despite experiencing an average of 8.9 attacks during this period [49]. Findings in ENABLE and other contemporary HAE cohorts indicate that access to effective LTP likely remains limited for many symptomatic patients, even in regions where first‐line therapies are available.

Before initiating lanadelumab, most patients reported perception of poor disease control, substantial HAE‐related impairment in HRQoL, above‐normal levels of anxiety and fatigue, and considerable impairment in work productivity and daily activities. These findings are consistent with the burden of HAE described by several large‐scale patient surveys across different regions [5, 50, 51]. Given pre‐lanadelumab attack characteristics were missing for most patients, and the lanadelumab treatment period was considerably longer than the pre‐lanadelumab period, it was considered inappropriate to directly compare attack characteristics before and after lanadelumab initiation.

Patient characteristics in ENABLE were comparable to other lanadelumab clinical studies [19, 20, 22] and are generally representative of patients with HAE in clinical practice [29, 30, 32, 33, 34]. Most enrolled patients (62.3%) were female, consistent with other HAE studies [7, 29, 30, 32, 33, 34], and a recent systematic literature review (5861/9122 [64.3%] patients with HAE with available data were female). Diagnostic delay in ENABLE was characterized by a right‐skewed distribution (mean > median [8.8 > 4.2 years]), similar to EMPOWER (7.3 > 2.5 years) [33], potentially attributed to longer delays in older patients. A 2018 survey of 250 patients reported considerably longer median diagnostic delay in patients born during 1950–1960 (7.0 years) than those born during 1980–1990 (1.4 years) [52]. We were interested to note that ENABLE included 7 patients aged ≥ 65 years at enrollment, since the views, needs, and goals of elderly and younger adults may differ, and treatment outcomes are not frequently reported [53].

In ENABLE, ∼30% of patients had ≥ 1 dosing‐interval extension from Q2W by Month 6, reaching ∼60% by Month 12, similar to INTEGRATED (29.4% and 56.4%) [34] and higher than in EMPOWER (24.0% and 28.9% of all patients) [33], possibly reflecting regional practice differences. The United States label recommends dosing‐interval extension to Q4W in patients well‐controlled on lanadelumab (e.g., attack‐free for > 6 months) [14]; the EU label recommends considering dosing‐interval extension to Q4W in stably attack‐free patients (no attack‐free timeframe specified) [15]. In a recent United States retrospective chart review of 75 lanadelumab‐treated patients, the median time to first dosing‐interval extension was 6.5 months [24]. In ENABLE, the proportion of patients with dosing‐interval extension to Q4W within 12 months was higher among those who enrolled after March 1, 2021 versus those who enrolled before. This different timing in dosing‐interval extension after lanadelumab initiation could be due to different reimbursement systems or treating physicians becoming more confident with increasing real‐world experience of lanadelumab in global clinical practice.

After 1 month of lanadelumab treatment, ∼80% of patients perceived their disease control to be adequate (AECT total score ≥ 10). Improved perception of disease control was maintained throughout the study. The proportion of patients reporting adequate disease control at Months 12 and 24 were comparable with that in HELP OLE [54], consolidating this result in real‐world practice. The mean baseline AE‐QoL total score in patients from ENABLE (42.9) was comparable with that in HELP (42.8–48.8) [55] and in newly enrolled patients in HELP OLE (∼40) [22]. Notably, clinically important improvement in HRQoL (≥ 6‐point decrease in AE‐QoL total score) [37, 38] was observed 1 month after initiating lanadelumab and sustained long‐term, as in previous studies [54, 55]. To the best of our knowledge, this is the first report of clinically meaningful HRQoL improvement observed as early as 1 month after initiating lanadelumab.

Consistent with AE‐QoL Fatigue/Mood domain score results, FSS results showed that most patients experienced clinically relevant fatigue before lanadelumab initiation, which improved substantially with lanadelumab treatment. Fatigue is a common but underrecognized HAE symptom [56, 57, 58], potentially a consequence of physiological and emotional stress from attacks. A recent study identified fatigue as the link between anxiety, work productivity, and activity impairment in HAE [58]. Although others have assessed fatigue via the AE‐QoL Fatigue/Mood domain [54, 55], ENABLE appears to be the first lanadelumab study to assess HAE‐related fatigue using a fatigue‐specific tool (FSS) [39]. Sustained improvements in other PROs, including health‐related loss of work productivity and activity impairment, and treatment satisfaction were also observed shortly after lanadelumab treatment. Although sample size gradually decreased over time, this trend was consistent with previous studies [54, 55].

ENABLE safety findings were generally consistent with HELP OLE and EMPOWER, both with > 2‐year follow‐up [22, 33]. As no lanadelumab‐related TEAEs resulted in treatment or study discontinuation in ENABLE, safety outcomes are unlikely to be influenced by drop‐out bias. The high cumulative number of 474 TEAEs occurring in 95 (68.8%) patients is expected given the long follow‐up; most were unrelated to lanadelumab treatment and none were fatal. Around 40% of patients experienced ≥ 1 infection/infestation‐type TEAE, which was expected for a study conducted during the COVID‐19 pandemic. Most treatment‐related TEAEs were mild ISRs. Although ISR incidence (11.6%) was higher than EMPOWER (none reported) [33], it was considerably lower than HELP OLE (42.5%) [22]. The importance of treatment adherence is highlighted by the single serious and severe treatment‐related TEAE of laryngeal edema, attributed to temporary discontinuation of lanadelumab by the patient.

As with any real‐world studies, limitations include potential recall biases, missing data, and inaccurate or delayed data capture. For instance, locations for 73% of on‐treatment physician‐reported attacks were missing/unknown, likely contributing to discrepancies between physician‐reported and patient‐reported data. Similarly, most responses to TSQM‐9 at Month 24 were not captured due to study site deployment issues. Moreover, as some patients were enrolled for up to 24 instead of 36 months, study findings could be confounded by sample‐size reductions between Months 24 and 36. Regarding discontinuations, several patients discontinued based on “patient's decision” or “physician's decision.” Further details were not requested in the eCRF, but follow‐up with investigators indicated reasons including non‐compliance with diary completion, co‐morbid mental health issues, and lack of efficacy. Additionally, 55.8% of patients were enrolled at 3 sites in Germany, Italy, and Switzerland, potentially reducing the generalizability of findings. Nevertheless, real‐world studies are invaluable for advancing the care of rare diseases such as HAE, as they enable the documentation of disease course and responses to interventions from a wide range of patients. Patient registries will similarly help to address knowledge gaps in HAE pathophysiology and management outcomes [59].

As lanadelumab was indicated only for patients aged ≥ 12 years during enrollment of ENABLE, which started in 2019, patients aged 2 to < 12 years were not enrolled. The 52‐week SPRING Study, which completed at the end of 2021, reported a 94.8% attack‐rate reduction with lanadelumab in patients aged 2 to < 12 years (mean monthly attack rate: 1.84 [baseline] vs. 0.08 [treatment period]) [23]. Future real‐world studies evaluating the long‐term effectiveness of lanadelumab in pediatric patients are warranted, further to approval in Europe, the United States, and other regions [12, 13, 14, 15, 23].

In conclusion, findings from ENABLE demonstrated the long‐term effectiveness of lanadelumab in clinical practice; substantial reductions in HAE attack rates and improvements in patient‐reported HRQoL were maintained for up to 36 months. The safety profile of lanadelumab was consistent with previous studies [19, 20, 21, 22] and no new safety signals were recorded. In line with previous Phase 3 trials [19, 20, 22, 54, 55], and recently published real‐world studies [33, 34], results from ENABLE support the benefits of lanadelumab for patients with HAE and its use as first‐line LTP, as recommended by the most recent HAE management guidelines [9, 17, 18].

## Author Contributions

**Andrea Zanichelli:** investigation, writing – original draft, writing – review and editing. **Walter A. Wuillemin:** investigation, writing – original draft, writing – review and editing. **Markus Magerl:** investigation, writing – original draft, writing – review and editing. **Andreas Recke:** investigation, writing – original draft, writing – review and editing. **Mauro Cancian:** investigation, writing – original draft, writing – review and editing. **Emel Aygören‐Pürsün:** investigation, writing – original draft, writing – review and editing. **Ramón Lleonart Bellfill:** investigation, writing – original draft, writing – review and editing. **Aharon Kessel:** investigation, writing – original draft, writing – review and editing. **Tamar Kinaciyan:** investigation, writing – original draft, writing – review and editing. **Robin Lochbaum:** investigation, writing – original draft, writing – review and editing. **Mona Al‐Ahmad:** investigation, writing – original draft, writing – review and editing. **Irmgard Andresen:** writing – original draft, writing – review and editing. **Maureen Watt:** conceptualization, methodology, validation, writing – original draft, writing – review and editing. **Natalie Khutoryansky:** data curation, formal analysis, validation, writing – original draft, writing – review and editing. **Daniel N. Castaner:** conceptualization, methodology, data curation, validation, writing – original draft, writing – review and editing. **Inmaculada Martinez‐Saguer:** investigation, writing – original draft, writing – review and editing. All authors approved the final version of the manuscript.

## Ethics Statement

The study protocol was approved by an independent institutional review board and site‐specific independent ethics committees, as applicable by local regulations. Patients or their legally authorized representatives provided written informed consent. The study was conducted in accordance with the International Conference on Harmonisation and Good Clinical Practice guidelines and the ethical principles outlined in the Declaration of Helsinki.

## Conflicts of Interest

A. Zanichelli has received speaker/consultancy fees from Astria Therapeutics, BioCryst, CSL Behring, KalVista Pharmaceuticals, Pharming, and Takeda. W. A. Wuillemin has received research grant support and speaker/consultancy fees from CSL Behring and Takeda. M. Magerl has received research grant support and/or speaker/consultancy fees from Argo, Astria Therapeutics, BioCryst, CSL Behring, Intellia Therapeutics, KalVista Pharmaceuticals, Octapharma, Otsuka, Pharvaris, and Takeda. A. Recke has received research grants from Deutsche Forschungsgemeinschaft and Euroimmun; travel and conference support from BioCryst, Novartis, Otsuka, Pharming, Stallergenes Greer, and Takeda; speaker honoraria from Bencard, BioCryst, CSL Behring, Euroimmun, KalVista, Novartis, Otsuka, and Takeda; and has served as a consultant or participated in advisory boards for BioCryst, CSL Behring, Novartis, Pharvaris, Swedish Orphan Biovitrum, and Takeda. M. Cancian has received personal fees from BioCryst, CSL Behring, KalVista Pharmaceuticals, Otsuka, Pharvaris, and Takeda. E. Aygören‐Pürsün has received honoraria, research funding, travel grants, and/or consulting fees from Astria Therapeutics, BioCryst, CSL Behring, Intellia Therapeutics, KalVista Pharmaceuticals, Otsuka, Pharvaris, and/or Takeda. R. Lleonart Bellfill has received speaker and/or consultancy fees, and has participated in advisory boards for BioCryst, CSL Behring, Novartis, Pharming, and Takeda; is or has been a clinical trial/registry investigator for BioCryst, IONIS, KalVista, Pharvaris, and Takeda; has received honoraria from BioCryst, CSL Behring, KalVista, Novartis, and Takeda, as well as funding to attend conferences and educational events from CSL Behring, Novartis, Pharming, and Takeda. A. Kessel has received travel grants from Pharming and Takeda; and honoraria from CSL Behring and Takeda. T. Kinaciyan has received research funding, speaker honoraria/consultancy fees, and travel grants from BioCryst, CSL Behring, KalVista Pharmaceuticals, Otsuka, Pharvaris, Sanofi, and Takeda. R. Lochbaum has received speaker/consultancy fees and/or travel grants from BioCryst, CSL Behring, KalVista Pharmaceuticals, Otsuka, and Takeda; and a research grant from Takeda. M. Al‐Ahmad reports speaker fees from AstraZeneca, GSK, Novartis, Sanofi, and Takeda. I. Andresen is an employee of Takeda Pharmaceuticals International AG and holds stock/options in Takeda Pharmaceutical Company Limited. M. Watt is an employee of Takeda Development Center Americas Inc. and holds stock/options in Takeda Pharmaceutical Company Limited. N. Khutoryansky is an employee of Cytel, contracted by Takeda Development Center Americas Inc. D. N. Castaner is an employee of Takeda Development Center Americas Inc. and holds stock/options in Takeda Pharmaceutical Company Limited. I. Martinez‐Saguer has received research funding support and/or speaker/consultant fees from BioCryst, CSL Behring, KalVista Pharmaceuticals, Octapharma, Pharming, Pharvaris, and Takeda/Shire.

## Supporting information


Supporting Information S1

## Data Availability

The dataset, including the redacted study protocol, redacted statistical analysis plan, and individual participants' data supporting the results reported in this article, will be made available within 3 months from initial request to researchers who provide a methodologically sound proposal. The data will be provided after its de‐identification, in compliance with applicable privacy laws, data protection, and requirements for consent and anonymization.
