# Supplementary material for: Sustained Effectiveness of Lanadelumab in Preventing Hereditary Angioedema Attacks: The ENABLE Study
Source: Clin Transl Allergy. 2026 Aug 6;16(8):e70190. doi: 10.1002/clt2.70190 (PMC13447952; doi:10.1002/clt2.70190)
Supplement: Supplementary file 1 [file CLT2-16-e70190-s001.pdf]

Supporting Information

Sustained effectiveness of lanadelumab in preventing hereditary angioedema attacks: The ENABLE Study

Andrea Zanichelli,[1,2] Walter A. Wuillemin,[3] Markus Magerl,[4,5] Andreas Recke,[6] Mauro Cancian,[7] Emel Aygören-Pürsün,[8] Ramón Lleonart Bellfill,[9] Aharon Kessel,[10] Tamar Kinaciyan,[11] Robin Lochbaum,[12] Mona Al-Ahmad,[13] Irmgard Andresen,[14] Maureen Watt,[15] Natalie Khutoryansky,[15] Daniel N. Castaner,[15] Inmaculada Martinez-Saguer[16]

[1]Operative Unit of Medicine, Angioedema Center, IRCCS Policlinico San Donato, San Donato Milanese, Milan, Italy; [2]Dipartimento di Scienze Biomediche per la Salute, University of Milan, Milan, Italy; [3]Luzerner Kantonsspital, Lucerne and University of Berne, Berne, Switzerland; [4]Charité-Universitätsmedizin Berlin (corporate member of Freie Universität Berlin and Humboldt-Universität zu Berlin), Berlin, Germany; [5]Fraunhofer Institute for Translational Medicine and Pharmacology ITMP IA, Berlin, Germany; [6]University of Lübeck, Lübeck, Germany; [7]University Hospital of Padua, Padua, Italy; [8]University Hospital Frankfurt, Goethe University Frankfurt, Frankfurt, Germany; [9]Allergology Department, Hospital Universitari de Bellvitge, Institut de Recerca IDIBELL, L'Hospitalet de Llobregat, Barcelona, Spain; [10]Bnai Zion Medical Centre, Technion Faculty of Medicine, Haifa, Israel; [11]Department of Dermatology, Medical University of Vienna, Vienna, Austria; [12]Ulm University Medical Center, Ulm, Germany; [13]Microbiology Department, College of Medicine, Kuwait University, Kuwait City, Kuwait; [14]Takeda Pharmaceuticals International AG, Zurich, Switzerland; [15]Takeda Development Center Americas, Inc., Lexington, MA, USA; [16]HZRM Hemophilia Center Rhine Main, Frankfurt/Main, Germany

ENABLE Study Investigators
Supplementary Methods
Supplementary Tables S1–S7
Supplementary Figures S1–S12

ENABLE Study Investigators

Nancy Agmon-Levin, Ramat-Gan, Israel.
Mona Al-Ahmad, Kuwait City, Kuwait.
Blanca Andrés-López, Barcelona, Spain.
Emel Aygören-Pürsün, Frankfurt, Germany.
Mauro Cancian, Padua, Italy.
Victoria Cardona, Barcelona, Spain.
Eva-Vanessa Ebert, Munich, Germany.
Antonio Gidaro, Milan, Italy.
Daniel Granados Pino, Barcelona, Spain.
Jens Greve, Ulm, Germany.
Mar Guilarte, Barcelona, Spain.
Karin Hartmann, Basel, Switzerland.
Felix Johnson, Innsbruck, Austria.
Aharon Kessel, Haifa, Israel.
Tamar Kinaciyan, Vienna, Austria.
Lisa Knipps, Düsseldorf, Germany.
Ramon Lleonart Bellfill, Barcelona, Spain.
Markus Magerl, Berlin, Germany.
Inmaculada Martinez-Saguer, Frankfurt/Main, Germany.
Marcus Maurer, Berlin, Germany.
Andreas Recke, Lübeck, Germany.
Antonia Scherer, Düsseldorf, Germany.
Giuseppe Spadaro, Naples, Italy.
Ulrich Strassen, Munich, Germany.
Martin Wagenmann, Düsseldorf, Germany.
Magdalena Wirth, Munich, Germany.
Walter Wuillemin, Berne, Switzerland.
Andrea Zanichelli, Milan, Italy.

Supplementary methods

Patient-reported outcome measures

AECT: The Angioedema Control Test (AECT) has a score of 0–16, where higher score indicates perception of greater disease control; the minimal clinically important difference is defined as changes of ≥3 points; <10 indicating perception of poor disease control and ≥10 indicating perception of adequate disease control.[1-3] AECT was administered via the same mobile application as the HAE attack diary at baseline, Months 1, 2, 3, 6, 12 and every 12 months thereafter until the end of follow-up.

AE-QoL: The Angioedema Quality of Life Questionnaire (AE-QoL) is a validated, angioedema-specific quality-of-life questionnaire assessing HRQoL impairment in patients with recurrent angioedema with a recall period of "within the last 4 weeks", comprised of 17 questions with 4 domain scores (Functioning, Fatigue/Mood, Fears/Shame, and Nutrition) and a total score.[4] The AE-QoL total and domain scores are transformed to a scale of 0–100, where higher scores indicate greater impairment. Clinically meaningful improvement in AE-QoL is defined as ≥6-point decrease in the AE-QoL total score. Moderate-to-large HRQoL impairment is represented by an AE-QoL total score of ≥39 points.[4-6] AE-QoL was administered via the same mobile application as the HAE attack diary at baseline, Months 1, 2, 3, 6, and every 6 months thereafter until the end of follow-up.

FSS: The Fatigue Severity Scale Questionnaire (FSS) is a validated, 9-item questionnaire measuring patients' fatigue severity and its impact on motivation, exercise, physical functioning, and work and social life.[7] It uses a 7-point Likert scale response (1 = strongly disagree, 7 = strongly agree), and the final score is obtained as mean of the response scores to the individual questions (ranges from 0–7).[7] Score ranges from 1–7 with higher score indicating worse fatigue; a mean FSS score of 2.3 is typical for healthy individuals and a mean score of ≥4 indicates clinically relevant fatigue.[7,8] FSS was administered via the same mobile application as the HAE attack diary at baseline, Months 1, 2, 3, 6, 12 and every 12 months thereafter until the end of follow-up.

HADS: The Hospital Anxiety and Depression Scale (HADS) can be used to assess levels of anxiety (7 items) or depression (7 items) over the past week.[9] Higher HADS subscale scores reflect more severe anxiety or depression (moderate: 11–14; severe, 15-21).[9,10] Higher overall HADS scores reflect greater psychological distress.[11] HADS was administered via the same mobile application as the HAE attack diary at baseline, Months 1, 2, 3, 6, 12 and every 12 months thereafter until the end of follow-up.

WPAI-GH: Work Productivity and Activity Impairment – General Health (WPAI-GH) is a 6-item assessment of the effect of health problems on patients' ability to work and perform regular activities, with a recall period of the last 7 days.[12,13] These items are used to calculate 4 scores with higher scores representing a greater loss of productivity: absenteeism (percent work time missed due to health), presenteeism (percent impairment while working due to health), overall work impairment due to health, and activity impairment due to health.[12,13] Scores are expressed as impairment percentages with higher scores indicating greater impairment and reduced productivity).[12,13] WPAI:GH was administered to patients aged ≥18 years via the same mobile application as the HAE attack diary at baseline, Months 1, 2, 3, 6, and every 6 months thereafter until the end of follow-up.

TSQM-9: The Treatment Satisfaction Questionnaire for Medication-9 (TSQM-9) consists of 9 items to evaluate 3 key domains: effectiveness, side effects, and convenience. Each item is rated on a 5- or 7-point Likert scale and scores transformed to a 0–100 scale for each domain, with higher

score indicating greater treatment satisfaction.[14] TSQM-9 was administered to patients aged ≥18 years via the same mobile application as the HAE attack diary at baseline, Months 1, 2, 3, 6, 12, and 36.

Supplementary Tables

TABLE S1. AECT total score over time

Visit	Baseline	Month 1	Month 2	Month 3	Month 6	Month 12	Month 24	Month 36
N	138	107	119	112	132	120	116	48
n	129	103	115	109	118	103	97	39
Mean (SD)	7.5 (3.71)	12.5 (3.55)	13.3 (3.36)	13.6 (3.56)	13.9 (3.25)	13.9 (3.06)	13.6 (3.42)	14.4 (2.85)
Median	7.0	13.0	15.0	15.0	16.0	16.0	15.0	16.0
Q1, Q3	5.0, 10.0	10.0, 16.0	12.0, 16.0	12.0, 16.0	12.0, 16.0	12.0, 16.0	12.0, 16.0	14.0, 16.0
Range	0–16	4–16	5–16	1–16	3–16	3–16	1–16	5–16

AECT score ranges from 0–16 with higher score indicating perception of better disease control; AECT score <10, perception of poorly controlled disease, AECT ≥10, perception of well-controlled disease; a change of 3 points is considered the minimal clinically important difference).[1-3] Baseline defined as the last observed value taken at enrollment assessment before lanadelumab initiation.

AECT, Angioedema Control Test; Q1, first quartile; Q3, third quartile; SD, standard deviation.

TABLE S2. AE-QoL scores over time

**Visit**	**Baseline**	**Month 1**	**Month 2**	**Month 3**	**Month 6**	**Month 12**	**Month 18**	**Month 24**	**Month 30**	**Month 36**
N	138	107	119	112	132	120	117	116	51	48
n	130	105	118	111	119	104	99	98	46	39
**Total score**										
Mean (SD)	42.9 (18.23)	29.1 (19.88)	27.1 (20.17)	24.4 (19.57)	23.2 (17.98)	21.4 (17.13)	22.5 (19.18)	22.7 (20.23)	21.3 (17.98)	19.3 (17.86)
Median	41.2	22.1	22.1	19.1	17.6	16.2	17.6	16.2	19.1	14.7
Q1, Q3	27.9, 57.4	14.7, 39.7	11.8, 41.2	10.3, 32.4	10.3, 32.4	10.3, 30.9	8.8, 33.8	7.4, 30.9	7.4, 32.4	7.4, 27.9
Range	9–82	0–82	0–81	0–84	0–88	0–79	0–87	0–93	0–84	0–71
**Functioning score**										
Mean (SD)	44.5 (24.78)	20.7 (24.50)	18.8 (24.64)	16.8 (22.91)	13.6 (22.69)	11.7 (19.21)	13.6 (20.69)	15.5 (21.84)	12.9 (17.70)	12.7 (19.42)
Median	50.0	12.5	6.3	6.3	0.0	0.0	0.0	0.0	0.0	0.0
Q1, Q3	25.0, 62.5	0.0, 31.3	0.0, 37.5	0.0, 25.0	0.0, 18.8	0.0, 18.8	0.0, 25.0	0.0, 25.0	0.0, 25.0	0.0, 25.0
Range	0–100	0–88	0–88	0–81	0–81	0–75	0–81	0–81	0–75	0–75
**Fatigue/Mood score**										
Mean (SD)	42.2 (23.91)	34.5 (23.77)	33.5 (24.37)	33.4 (23.64)	33.4 (22.92)	33.2 (24.54)	33.7 (25.97)	31.9 (24.00)	32.9 (26.57)	29.2 (21.66)
Median	40.0	30.0	27.5	30.0	30.0	30.0	30.0	25.0	30.0	25.0
Q1, Q3	25.0, 60.0	20.0, 45.0	15.0, 50.0	15.0, 45.0	15.0, 50.0	15.0, 50.0	10.0, 50.0	10.0, 45.0	10.0, 50.0	15.0, 45.0
Range	0–100	0–100	0–100	0–100	0–100	0–100	0–95	0–100	0–100	0–80
**Fears/Shame score**										
Mean (SD)	46.2 (22.06)	33.5 (23.65)	30.4 (23.30)	25.3 (22.40)	24.1 (20.59)	21.1 (20.07)	21.8 (22.31)	22.4 (23.84)	20.0 (21.88)	17.2 (22.18)
Median	47.9	25.0	29.2	16.7	20.8	16.7	16.7	16.7	12.5	8.3
Q1, Q3	29.2, 62.5	16.7, 50.0	12.5, 45.8	8.3, 37.5	8.3, 33.3	4.2, 29.2	4.2, 33.3	4.2, 33.3	0.0, 33.3	0.0, 25.0
Range	0–96	0–92	0–100	0–92	0–92	0–92	0–92	0–100	0–92	0–79
**Food score**										
Mean (SD)	31.5 (25.20)	18.9 (24.95)	18.1 (25.87)	14.3 (22.35)	14.2 (21.77)	12.3 (19.71)	14.6 (21.95)	15.4 (25.12)	12.8 (20.83)	14.4 (21.75)
Median	25.0	12.5	12.5	0.0	0.0	0.0	0.0	0.0	0.0	0.0
Q1, Q3	12.5, 50.0	0.0, 25.0	0.0, 25.0	0.0, 25.0	0.0, 25.0	0.0, 25.0	0.0, 25.0	0.0, 25.0	0.0, 25.0	0.0, 25.0
Range	0–100	0–100	0–100	0–100	0–88	0–100	0–100	0–100	0–75	0–75

AE-QoL total score and domain scores are transformed to a scale of 0–100 with higher scores indicating greater impairment. Total score of 0–23 indicates no effect, 24–38 a small effect, and ≥39 a moderate-to-large effect of recurrent angioedema on patients' HRQoL; a change of 6 points is considered the minimal clinically important difference.[4,5]
AE-QoL, Angioedema Quality of Life Questionnaire; Q1, first quartile; Q3, third quartile; SD, standard deviation.

TABLE S3. FSS score over time

Visit	Baseline	Month 1	Month 2	Month 3	Month 6	Month 12	Month 24	Month 36
N	138	107	119	112	132	120	116	48
n	130	103	117	110	115	105	97	39
Mean (SD)	4.4 (1.54)	3.8 (1.70)	3.6 (1.61)	3.4 (1.58)	3.5 (1.62)	3.3 (1.62)	3.4 (1.60)	2.9 (1.57)
Median	4.5	3.8	3.7	3.2	3.3	3.1	3.3	2.3
Q1, Q3	3.0, 5.6	2.2, 5.3	2.3, 5.0	2.1, 4.7	2.1, 4.7	2.0, 4.4	2.0, 4.4	1.6, 4.4
Range	1–7	1–7	1–7	1–7	1–7	1–7	1–7	1–6

FSS scale score: Score ranges from 1–7 with higher score indicating worse fatigue; a score of 2.3 is typical for healthy individuals and a score of ≥4 indicates clinically relevant fatigue.[7,8]

FSS, Fatigue Severity Scale; Q1, first quartile; Q3, third quartile; SD, standard deviation.

TABLE S4. HADS scores over time

Visit	Baseline	Month 1	Month 2	Month 3	Month 6	Month 12	Month 24	Month 36
N	138	107	119	112	132	120	116	48
n	130	103	115	108	116	102	97	39
**Anxiety Subscale score**								
Mean (SD)	7.6 (4.32)	5.9 (4.24)	6.2 (5.18)	5.4 (4.25)	5.5 (4.29)	5.3 (4.41)	5.2 (4.43)	4.7 (4.39)
Median	8.0	5.0	5.0	5.0	5.0	5.0	4.0	4.0
Q1, Q3	4.0, 10.0	3.0, 8.0	2.0, 10.0	2.0, 8.0	2.0, 8.0	2.0, 8.0	2.0, 8.0	1.0, 7.0
Range	0–17	0–19	0–21	0–20	0–20	0–20	0–20	0–18
**Depression Subscale score**								
Mean (SD)	5.3 (3.98)	4.5 (4.13)	4.5 (4.52)	4.2 (4.27)	4.0 (3.99)	3.8 (4.08)	4.0 (4.09)	3.2 (3.67)
Median	5.0	4.0	3.0	3.0	3.0	2.0	3.0	2.0
Q1, Q3	2.0, 8.0	1.0, 6.0	1.0, 7.0	1.0, 6.0	1.0, 6.0	1.0, 6.0	1.0, 6.0	0.0, 6.0
Range	0–18	0–17	0–21	0–17	0–18	0–18	0–16	0–14

HADS subscale scores (scores range from 0–21 with higher scores indicating greater impairment: scores of 0–7 indicate normal levels of anxiety or depression, 8–10 mild, 11–14 moderate, and 15–21 severe anxiety or depression).[9,10]
HADS, Hospital Anxiety and Depression Scale; Q1, first quartile; Q3, third quartile; SD, standard deviation.

TABLE S5. WPAI:GH scores over time

Visit	Baseline	Month 1	Month 2	Month 3	Month 6	Month 12	Month 18	Month 24	Month 30	Month 36
N	130	100	113	106	124	114	111	110	47	44
**Absenteeism**										
n	82	60	75	73	78	67	69	63	28	22
Mean (SD)	10.6 (18.26)	4.8 (16.77)	7.9 (19.8)	9.4 (20.9)	6.8 (17.85)	4.8 (12.85)	10.0 (25.17)	5.3 (14.64)	5.7 (16.87)	0.0 (0.0)
Median	0.0	0.0	0.0	0.0	0.0	0.0	0.0	0.0	0.0	0.0
Q1, Q3	0.0, 16.7	0.0, 0.0	0.0, 0.0	0.0, 0.0	0.0, 0.0	0.0, 0.0	0.0, 0.0	0.0, 0.0	0.0, 0.0	0.0, 0.0
Range	0–100	0–100	0–91	0–100	0–75	0–50	0–100	0–50	0–60	0–0
**Presenteeism**										
n	81	59	75	72	78	67	66	63	28	22
Mean (SD)	35.7 (29.02)	17.8 (26.40)	18.5 (25.72)	17.6 (26.93)	12.7 (20.99)	15.5 (24.82)	13.2 (20.99)	16.8 (24.88)	12.1 (20.25)	7.7 (17.44)
Median	30.0	10.0	10.0	0.0	0.0	0.0	0.0	0.0	0.0	0.0
Q1, Q3	10.0, 60.0	0.0, 20.0	0.0, 30.0	0.0, 20.0	0.0, 20.0	0.0, 20.0	0.0, 20.0	0.0, 30.0	0.0, 20.0	0.0, 0.0
Min, Max	0, 100	0, 100	0, 100	0, 100	0, 70	0, 100	0, 80	0, 100	0, 80	0, 70
**Overall work productivity**										
n	81	59	75	72	78	67	66	63	28	22
Mean (SD)	39.5 (31.55)	19.0 (27.67)	22.6 (30.21)	22.4 (30.43)	17.4 (26.22)	18.5 (27.56)	16.6 (25.80)	20.5 (27.48)	16.4 (24.00)	7.7 (17.44)
Median	32.5	10.0	10.0	10.0	0.0	0.0	0.0	0.0	10.0	0.0
Q1, Q3	10.0, 70.0	0.0, 20.0	0.0, 30.0	0.0, 48.0	0.0, 20.0	0.0, 30.0	0.0, 20.0	0.0, 50.0	0.0, 20.0	0.0, 0.0
Range	0–100	0–100	0–100	0–100	0–88	0–100	0–92	0–100	0–88	0–70
**Activity impairment**										
n	120	94	107	104	109	96	95	91	42	33
Mean (SD)	41.3 (28.9)	27.1 (30.5)	24.6 (28.23)	23.6 (28.99)	19.1 (25.98)	19.2 (25.62)	18.3 (24.65)	24.4 (29.67)	19.0 (21.62)	11.8 (17.22)
Median	40.0	10.0	10.0	10.0	10.0	10.0	10.0	10.0	10.0	0.0
Q1, Q3	15.0, 70.0	0.0, 50.0	0.0, 50.0	0.0, 40.0	0.0, 20.0	0.0, 30.0	0.0, 30.0	0.0, 50.0	0.0, 30.0	0.0, 20.0
Range	0–100	0–100	0–100	0–90	0–90	0–90	0–90	0–100	0–80	0–70

WPAI:GH work productivity loss and activity impairment (scores are expressed as impairment percentages with higher scores indicating greater impairment and reduced productivity).[12,13]
Q1, first quartile; Q3, third quartile; SD, standard deviation; WPAI:GH, Work Productivity and Activity Impairment: General Health.

TABLE S6. TSQM-9 scores over time

Visit	Baseline	Month 1	Month 2	Month 3	Month 6	Month 12	Month 36
N	130	100	113	106	124	114	44
n	120	95	106	101	108	97	35
**Global satisfaction score**							
Mean (SD)	68.2 (21.50)	77.7 (22.60)	80.9 (19.18)	80.9 (21.60)	84.3 (19.53)	86.6 (17.65)	93.7 (13.73)
Median	69.4	76.4	84.0	84.7	91.7	91.7	100.0
Q1, Q3	52.8, 84.0	61.1, 100.0	75.0, 100.0	69.4, 100.0	76.4, 100.0	76.4, 100.0	91.7, 100.0
Range	0–100	7–100	13–100	0–100	22–100	29–100	29–100
**Effectiveness score**							
Mean (SD)	64.7 (21.90)	77.8 (26.55)	76.5 (30.62)	80.4 (27.35)	82.9 (27.26)	79.0 (32.19)	79.8 (36.18)
Median	66.7	83.3	88.9	94.4	94.4	94.4	100.0
Q1, Q3	50.0, 77.8	66.7, 100.0	66.7, 100.0	66.7, 100.0	80.6, 100.0	77.8, 100.0	83.3, 100.0
Range	0–100	0–100	0–100	0–100	0–100	0–100	0–100
**Convenience score**							
Mean (SD)	65.1 (21.63)	79.5 (18.97)	82.1 (17.92)	82.6 (17.27)	82.7 (17.33)	84.6 (16.52)	86.8 (19.81)
Median	66.7	83.3	83.3	88.9	83.3	88.9	100.0
Q1, Q3	50.0, 83.3	66.7, 100.0	66.7, 100.0	66.7, 100.0	72.2, 100.0	77.8, 100.0	72.2, 100.0
Range	17–100	17–100	22–100	39–100	28–100	28–100	28–100

TSQM-9 scores range from 0–100 with higher scores indicating greater satisfaction.[14] Data not shown for month 24 as TSQM-9 scores for most patients were not collected due to deployment issue.

Q1, first quartile; Q3, third quartile; SD, standard deviation; TSQM-9, Treatment Satisfaction Questionnaire for Medication-9.

TABLE S7. TEAEs that occurred in ≥5% of patients, by system organ class, preferred term both overall and by relationship to lanadelumab

Safety set (*N* = 138)<br>Patients with events (*n* = 95)<br>Events (*n* = 474)				
**System organ class**<br>Preferred term[†]	Overall		Related	
	Patients, *n* (%)	Events, *n* (%)	Patients, *n* (%)	Events, *n* (%)
**Any TEAE**	**95 (68.8)**	**474 (100)**	**25 (18.1)**	**84 (17.7)**
**Infections and infestations**				
COVID-19	39 (28.3)	44 (9.3)	0	0
Nasopharyngitis	13 (9.4)	16 (3.4)	0	0
**General disorders and administration site conditions**				
Fatigue	7 (5.1)	8 (1.7)	2 (1.4)	2 (0.4)
Injection-site erythema	7 (5.1)	18 (3.8)	7 (5.1)	18 (3.8)
**Gastrointestinal disorders**				
Toothache	7 (5.1)	11 (2.3)	0	0
**Nervous system disorders**				
Headache	14 (10.1)	22 (4.6)	2 (1.4)	2 (0.4)
**Musculoskeletal and connective tissue disorders**				
Arthralgia	7 (5.1)	21 (4.4)	0	0
Back pain	7 (5.1)	12 (2.5)	1 (0.7)	1 (0.2)
**Vascular disorders**				
Hypertension	10 (7.2)	10 (2.1)	0	0

[†]Adverse events were classified into system organ class and preferred term using version 27.0 of the Medical Dictionary for Regulatory Activities.
HAE, hereditary angioedema; TEAE, treatment-emergent adverse event.

Supplementary Figures

FIGURE S1. ENABLE Study design.

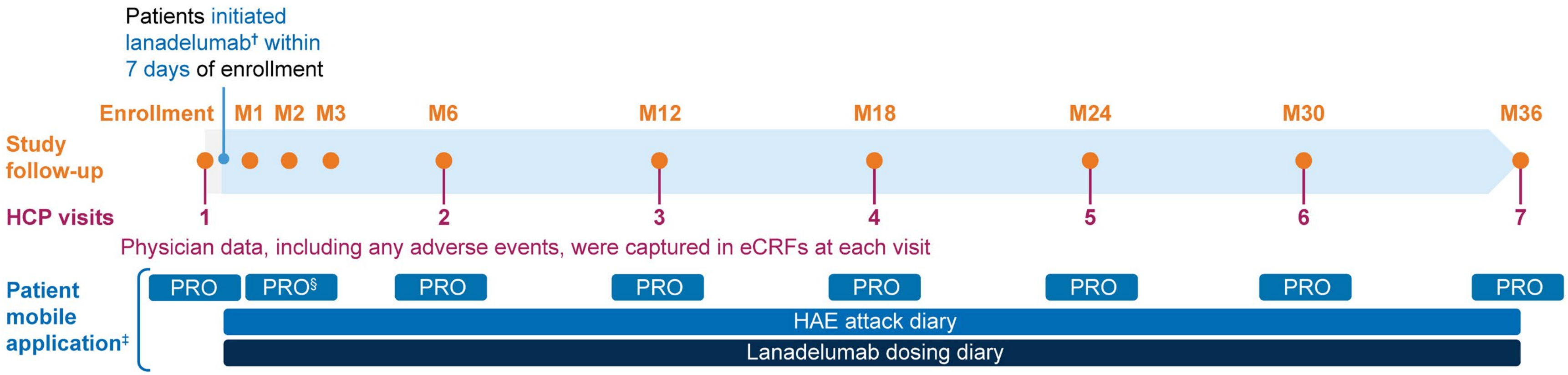

†Per approved label. ‡Patients recorded details on HAE attack and lanadelumab dosing with a smartphone application, which also sent out questionnaires to patients for collection of PROs at specific time points. §PRO questionnaires were administered in Months 1, 2, and 3.

HAE, hereditary angioedema; HCP, healthcare professional; M, Month; PRO, patient-reported outcome.

FIGURE S2. Patient disposition.

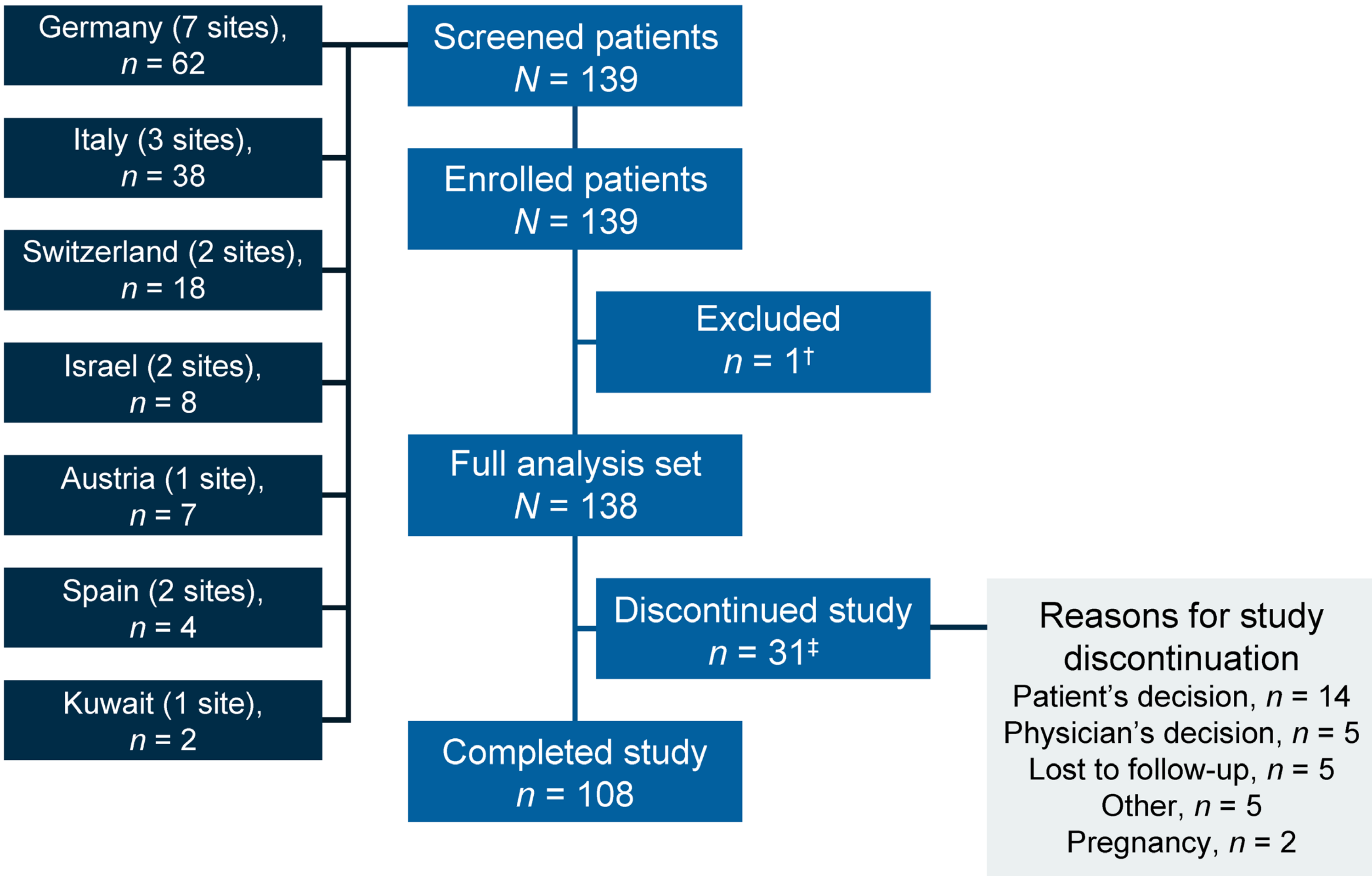

[†]Enrollment of 1 patient from Germany was stopped by sponsor after ePRO issue. [‡]Inclusive of the patient excluded from the full analysis set.

FIGURE S3. (A) Mean AECT total score from baseline to 6 months after lanadelumab initiation. (B) Median (Q1–Q3) AECT total score over time.

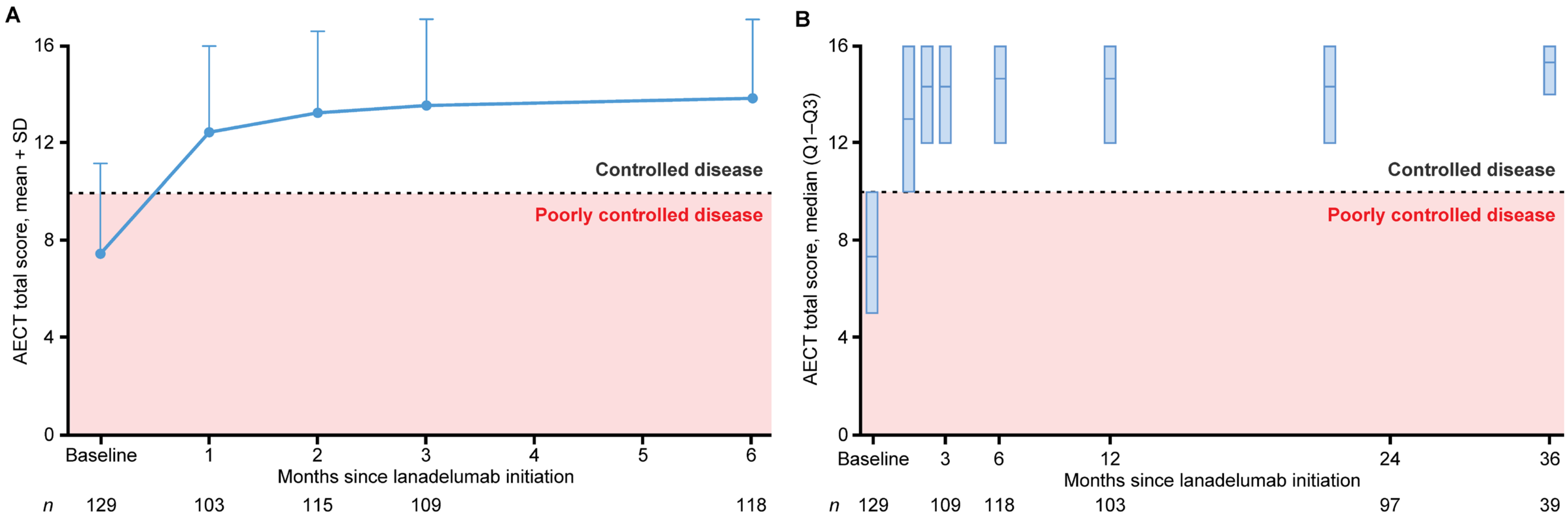

AECT total score ranges from 0 to 16 with higher score indicating perception of better disease control. A score of ≥10 indicates perception of well-controlled disease.[1,2] Baseline defined as the last observed value taken at enrollment assessment before lanadelumab initiation. Individual AECT total score values are reported in Table S1.
AECT, Angioedema Control Test; Q1, first quartile; Q3, third quartile; SD, standard deviation.

FIGURE S4. (A) Mean and (B) median AE-QoL total score from baseline to 6 months after lanadelumab initiation.

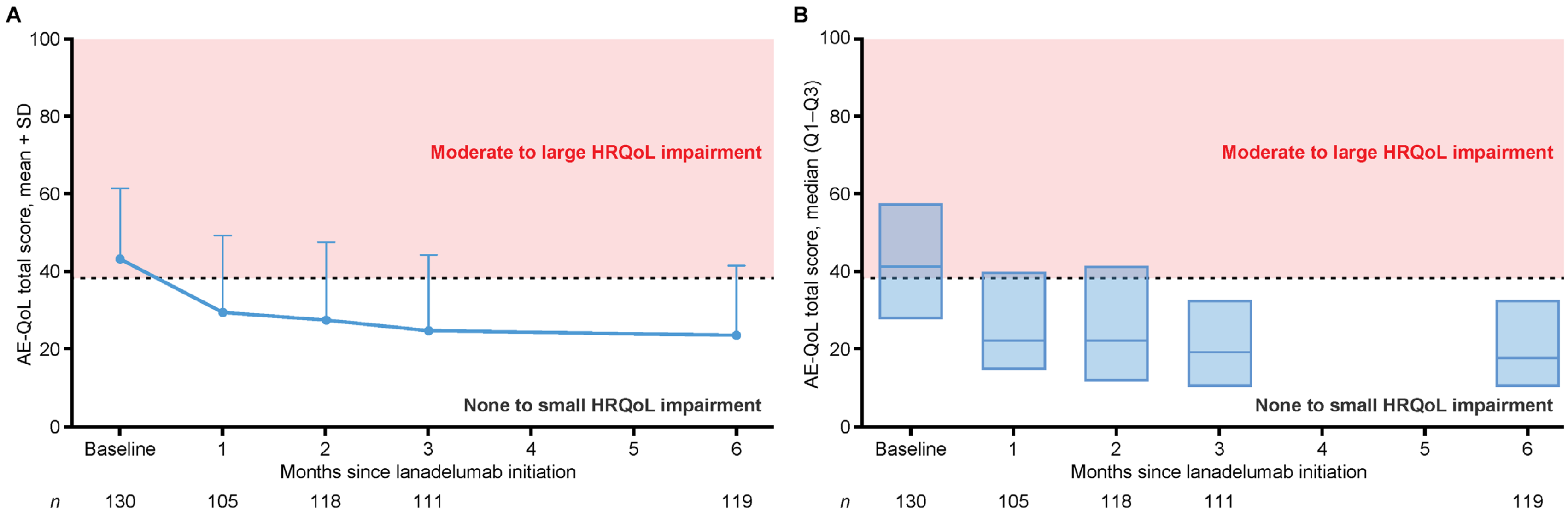

AE-QoL total score is transformed to a scale of 0–100 with higher score indicating greater impairment: a score of 0–23 indicates no effect, 24–38 a small effect, and ≥39 a moderate-to-large effect of recurrent angioedema on patients' HRQoL; a change of 6 points is considered the minimal clinically important difference.[4,5] Baseline defined as the last observed value taken at enrollment assessment before lanadelumab initiation. Individual values for AE-QoL total score are reported in Table S2.
AE-QoL, Angioedema Quality of Life Questionnaire; HRQoL, health-related quality of life; Q1, first quartile; Q3, third quartile; SD, standard deviation.

FIGURE S5. Mean AE-QoL Domain Scores of (A) Function, (B) Fatigue/mood, (C) Fear/shame, and (D) Nutrition during the study period.

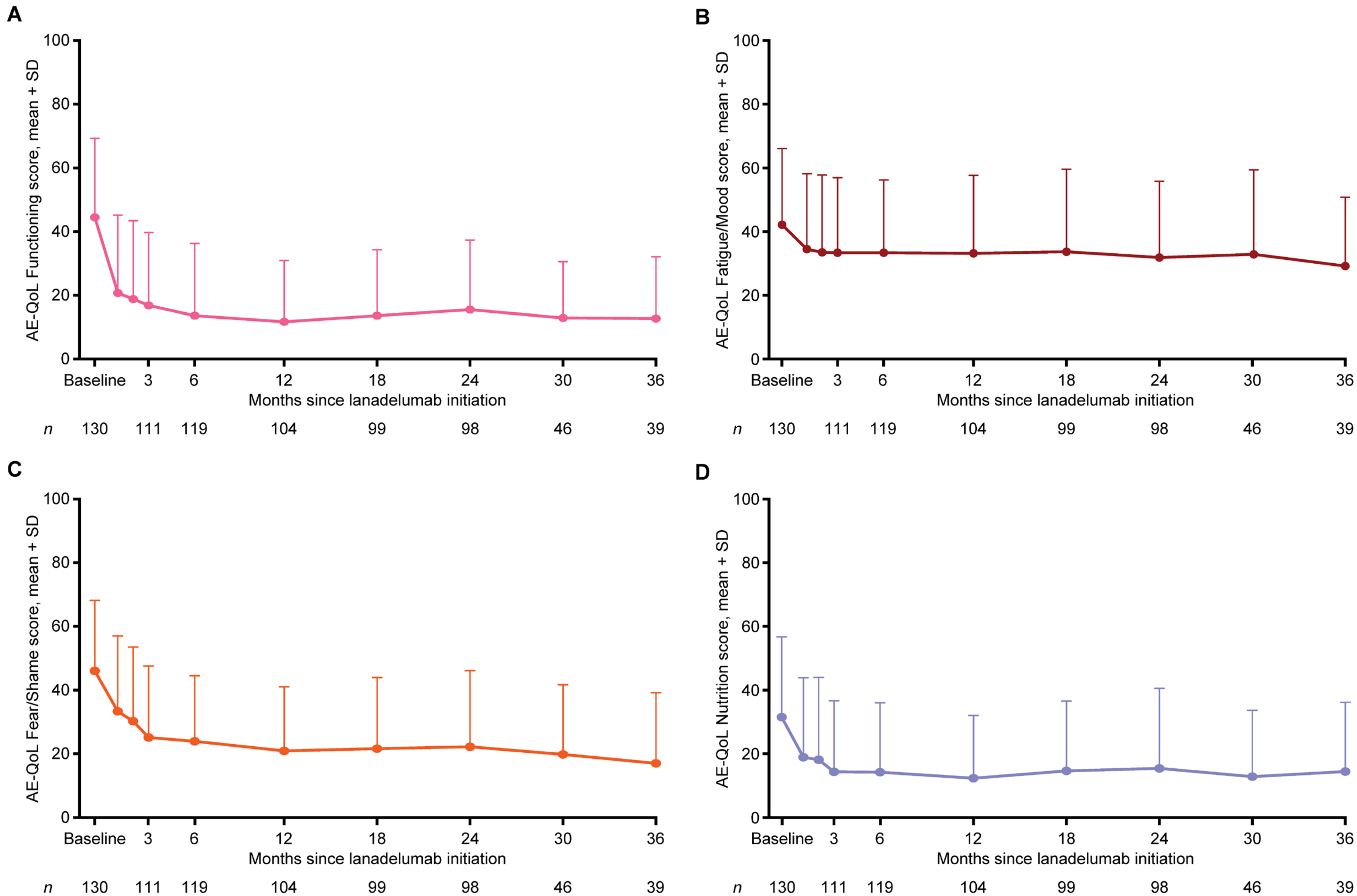

AE-QoL domain scores are transformed to a scale of 0–100, with lower scores indicating less impairment. Baseline defined as the last observed value taken at enrollment assessment before lanadelumab initiation. Individual values for AE-QoL domain scores are reported in Table S2.

AE-QoL, Angioedema Quality of Life Questionnaire; Q1, first quartile; Q3, third quartile; SD, standard deviation.

FIGURE S6. (A) Mean and (B) median FSS score from baseline to 6 months after lanadelumab initiation.

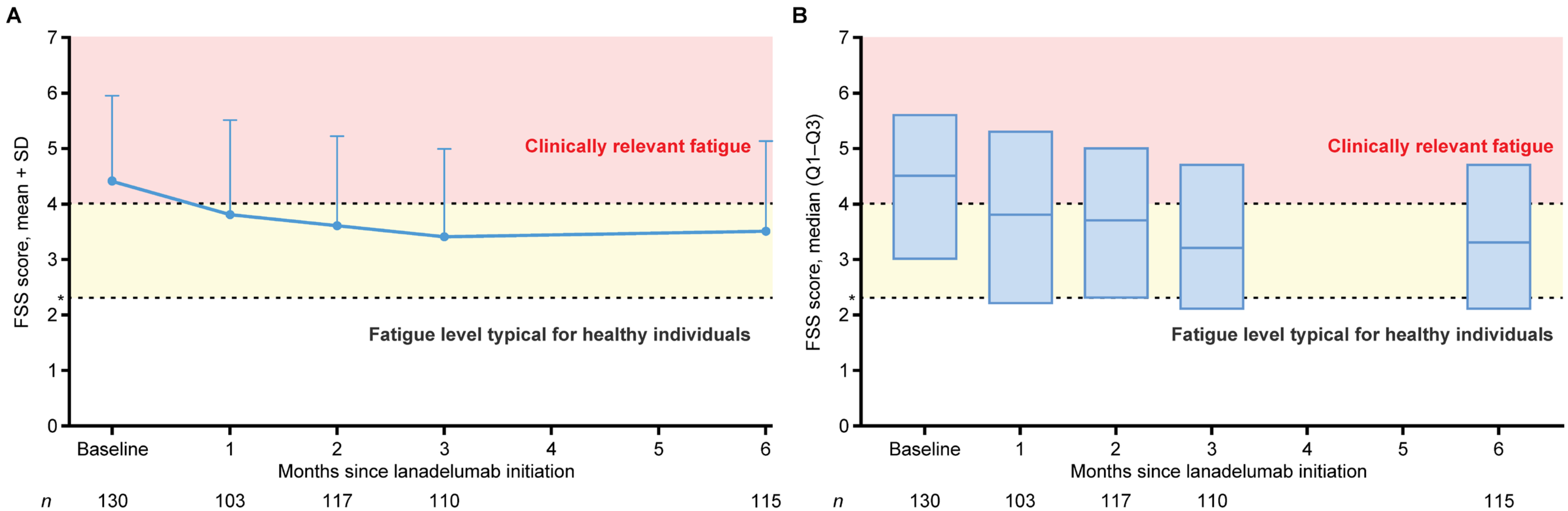

FSS score ranges from 1–7 with higher score indicating worse fatigue; a score of 2.3 (indicated with *) is typical for healthy individuals and a score of ≥4 indicates clinically relevant fatigue.[7,8] Baseline defined as the last observed value taken at enrollment assessment before lanadelumab initiation. Individual values of FSS scores are reported in Table S3.
FSS, Fatigue Severity Scale; Q1, first quartile; Q3, third quartile; SD, standard deviation.

FIGURE S7. (A) Mean and (B) median HADS subscale scores from baseline to 6 months after lanadelumab initiation.

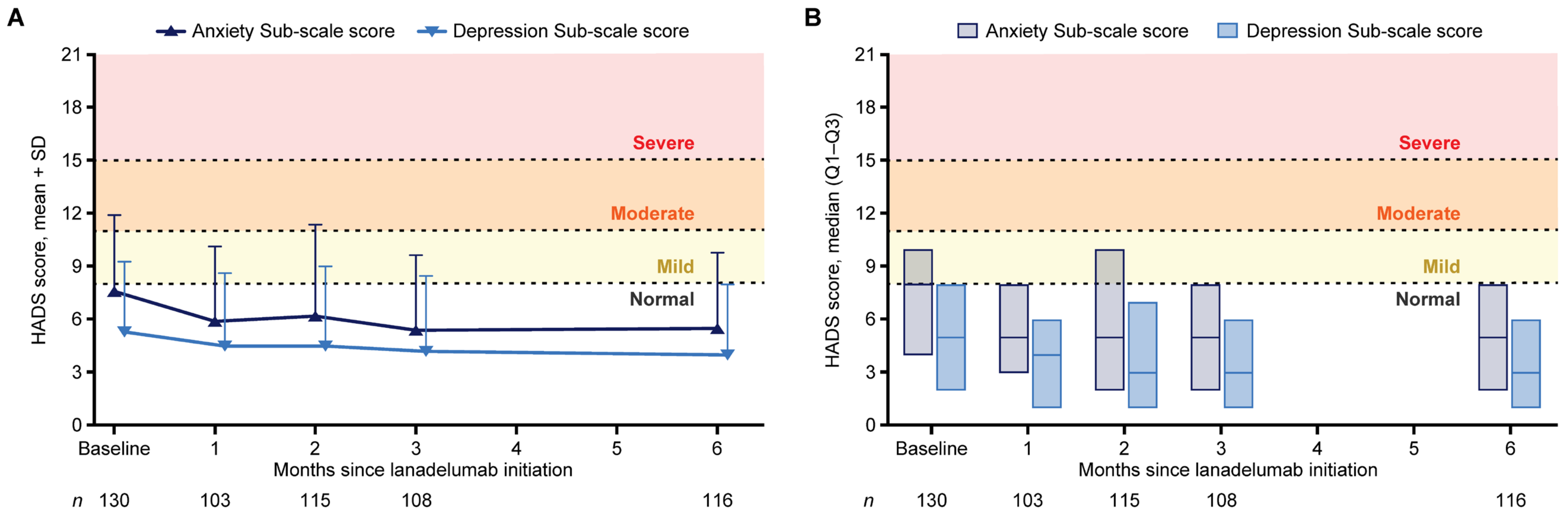

HADS scores range from 0 to 21 with higher score indicating greater impairment: scores of 0–7 indicates normal levels of anxiety or depression, 8–10 mild, 11–14 moderate, and 15–21 severe anxiety or depression.[9-11] Baseline defined as the last observed value taken at enrollment assessment before lanadelumab initiation. Individual values of HADS scores are reported in Table S4.

HADS, Hospital Anxiety and Depression Scale; Q1, first quartile; Q3, third quartile; SD, standard deviation.

FIGURE S8. (A) Mean and (B) median WPAI:GH scores for work productivity loss and activity impairment from baseline to 6 months after lanadelumab initiation.

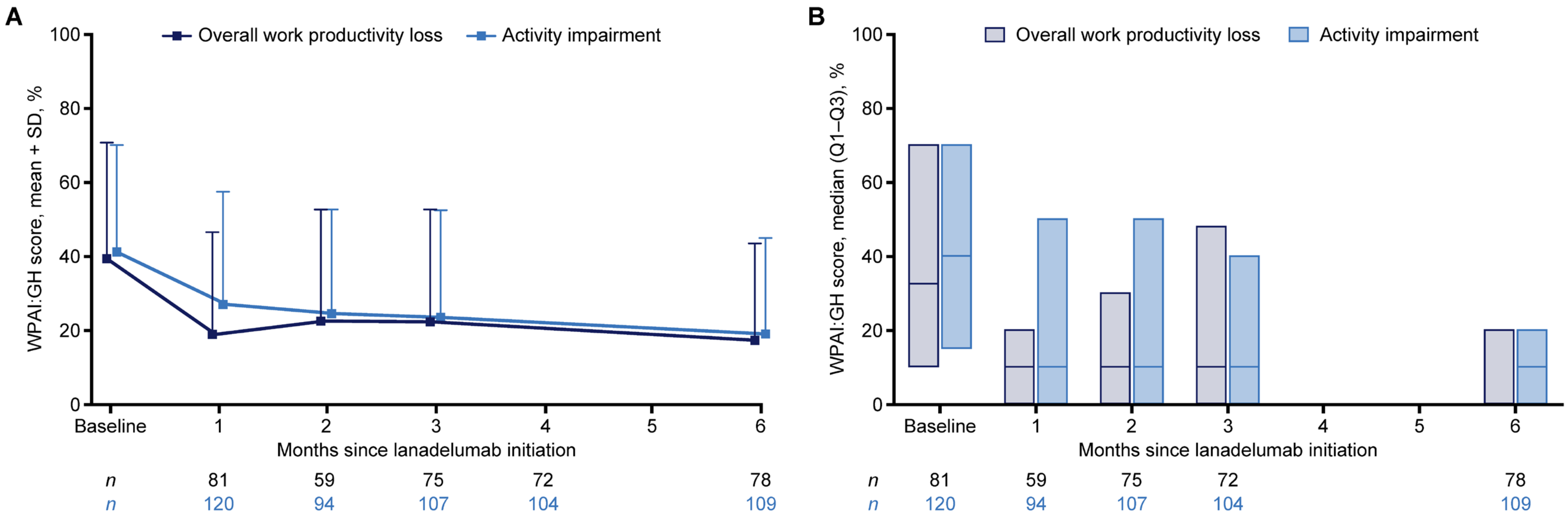

WPAI:GH scores are expressed as percentages of overall work productivity loss (combined absenteeism plus presenteeism) and activity impairment (impairment in regular daily nonwork activities), with higher scores indicating greater impairment.[12,13] Baseline defined as the last observed value taken at enrollment assessment before lanadelumab initiation. Individual WPAI:GH scores are reported in Table S5.

Q1, first quartile; Q3, third quartile; SD, standard deviation; WPAI:GH, Work Productivity and Activity Impairment: General Health.

FIGURE S9. Mean WPAI:GH scores for (A) Absenteeism (percentage of work time missed due to health) and (B) Presenteeism (percentage of impairment while working due to health) during the study period.

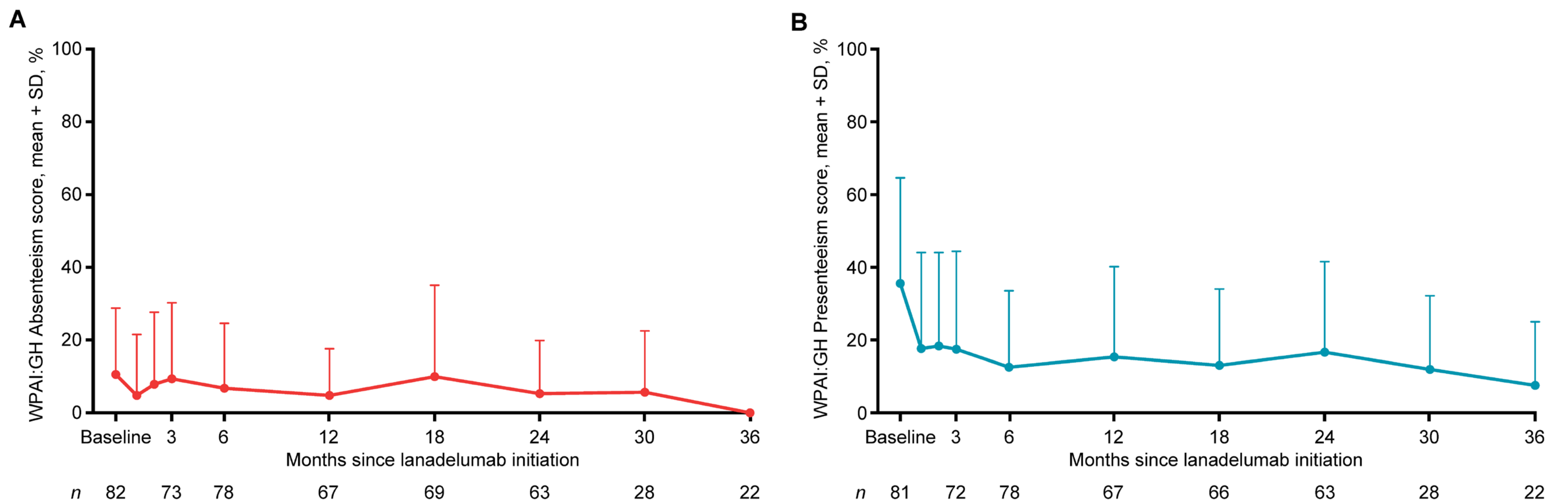

WPAI:GH scores are expressed as percentages of absenteeism (work time missed) and presenteeism (reduced productivity while working), with higher scores indicating greater impairment.[12,13] Baseline defined as the last observed value taken at enrollment assessment before lanadelumab initiation.
SD, standard deviation; WPAI:GH, Work Productivity and Activity Impairment: General Health.

FIGURE S10. (A) Mean and (B) median TSQM-9 Global Satisfaction Score from baseline to 6 months after lanadelumab initiation.

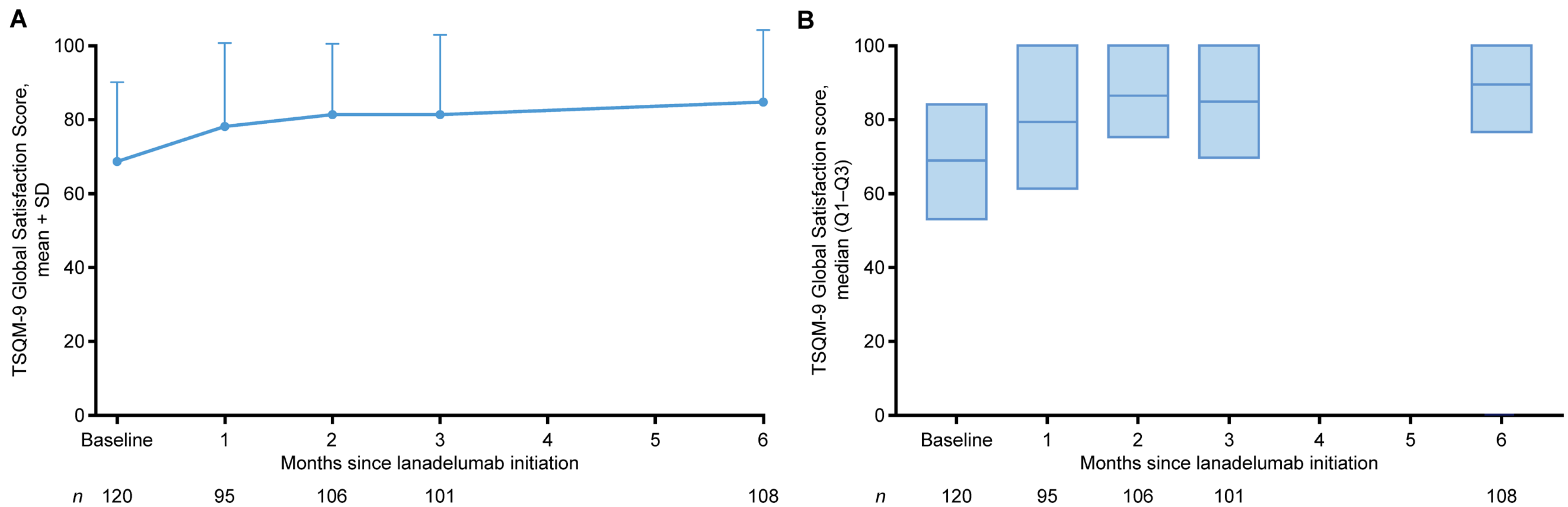

TSQM-9 item is rated on a 5- or 7-point Likert scale and scores transformed to a 0–100 scale for each domain, with higher scores indicating greater treatment satisfaction.[14] Baseline defined as the last observed value taken at enrollment assessment before lanadelumab initiation. Individual TSQM-9 scores are reported in Table S6.
Q1, first quartile; Q3, third quartile; SD, standard deviation; TSQM-9, Treatment Satisfaction Questionnaire for Medication-9.

FIGURE S11. Mean TSQM-9 (A) Convenience and (B) Effectiveness Scores during the study period.

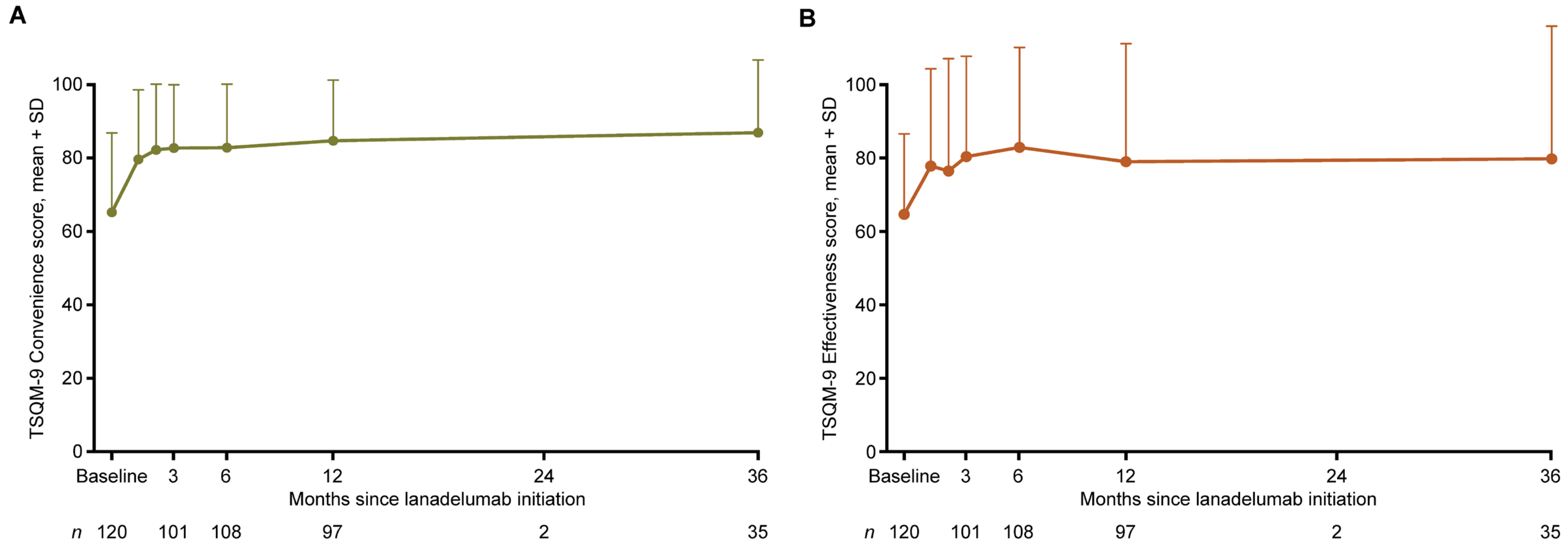

Each TSQM-9 item is rated on a 5- or 7-point Likert scale and scores transformed to a 0–100 scale for each domain; higher scores indicate greater treatment satisfaction.[14] Baseline defined as the last observed value taken at enrollment assessment before lanadelumab initiation. Individual TSQM-9 scores are reported in Table S6. Data not shown for month 24 as data were not captured for most patients due to deployment issues.

SD, standard deviation; TSQM-9, Treatment Satisfaction Questionnaire for Medication-9.

FIGURE S12. Incidence of lanadelumab-related injection-site reactions during the study period.

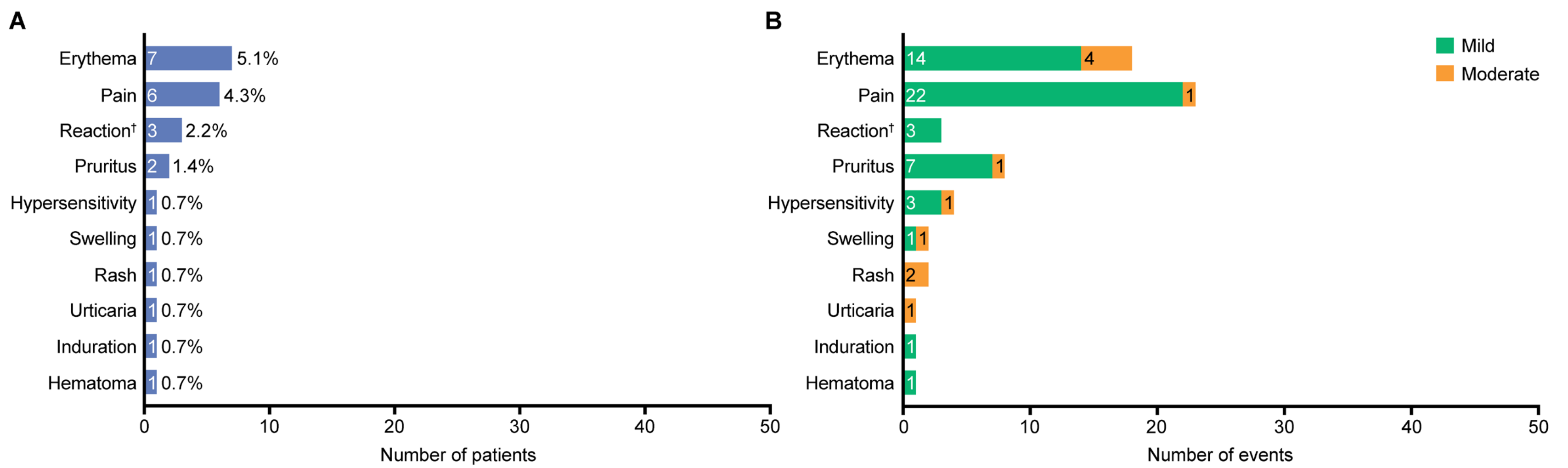

Panel A, number in each bar indicates the number of patients and percentage next to each bar indicates the percentage of patients (denominator=safety set) who had experienced the specific injection-site reaction event. Panel B, number at the base of each bar indicates the total number of specific injection-site reaction events experienced by all patients in the safety set. †Preferred terms: injection-site reaction (mild) in 2 patients (1 event each) and injection-related reaction (mild) in 1 patient with 1 event.

References

1. Weller K, Donoso T, Magerl M, et al. Development of the Angioedema Control Test-A patient-reported outcome measure that assesses disease control in patients with recurrent angioedema. *Allergy.* 2020;75(5):1165-1177.
2. Weller K, Donoso T, Magerl M, et al. Validation of the Angioedema Control Test (AECT)-A patient-reported outcome instrument for assessing angioedema control. *J Allergy Clin Immunol Pract.* 2020;8(6):2050-2057 e2054.
3. Fijen LM, Vera C, Buttgereit T, et al. Sensitivity to change and minimal clinically important difference of the angioedema control test. *Clin Transl Allergy.* 2023;13(9):e12295.
4. Weller K, Groffik A, Magerl M, et al. Development and construct validation of the angioedema quality of life questionnaire. *Allergy.* 2012;67(10):1289-1298.
5. Weller K, Magerl M, Peveling-Oberhag A, Martus P, Staubach P, Maurer M. The Angioedema Quality of Life Questionnaire (AE-QoL) - assessment of sensitivity to change and minimal clinically important difference. *Allergy.* 2016;71(8):1203-1209.
6. Kulthanan K, Chularojanamontri L, Rujitharanawong C, Weerasubpong P, Maurer M, Weller K. Angioedema quality of life questionnaire (AE-QoL) - Interpretability and sensitivity to change. *Health Qual Life Outcomes.* 2019;17(1):160.
7. Krupp LB, LaRocca NG, Muir-Nash J, Steinberg AD. The Fatigue Severity Scale. Application to patients with multiple sclerosis and systemic lupus erythematosus. *Arch Neurol.* 1989;46(10):1121-1123.
8. Valko PO, Bassetti CL, Bloch KE, Held U, Baumann CR. Validation of the fatigue severity scale in a Swiss cohort. *Sleep.* 2008;31(11):1601-1607.
9. Zigmond AS, Snaith RP. The Hospital Anxiety and Depression Scale. *Acta Psychiatr Scand.* 1983;67(6):361-370.
10. Stern AF. The Hospital Anxiety and Depression Scale. *Occup Med (Lond).* 2014;64(5):393-394.
11. Hyland KA, Hoogland AI, Gonzalez BD, et al. Evaluation of the psychometric and structural properties of the Spanish version of the Hospital Anxiety and Depression Scale in Latina cancer patients. *J Pain Symptom Manage.* 2019;58(2):289-296 e282.
12. Gawlicki MC, Reilly MC, Popielnicki A, Reilly K. Linguistic validation of the US Spanish work productivity and activity impairment questionnaire, general health version. *Value Health.* 2006;9(3):199-204.
13. Reilly MC, Zbrozek AS, Dukes EM. The validity and reproducibility of a work productivity and activity impairment instrument. *Pharmacoeconomics.* 1993;4(5):353-365.
14. Bharmal M, Payne K, Atkinson MJ, Desrosiers MP, Morisky DE, Gemmen E. Validation of an abbreviated Treatment Satisfaction Questionnaire for Medication (TSQM-9) among patients on antihypertensive medications. *Health Qual Life Outcomes.* 2009;7:36.
